# An organoid biobank for childhood kidney cancers that captures disease and tissue heterogeneity

**DOI:** 10.1038/s41467-020-15155-6

**Published:** 2020-03-11

**Authors:** Camilla Calandrini, Frans Schutgens, Rurika Oka, Thanasis Margaritis, Tito Candelli, Luka Mathijsen, Carola Ammerlaan, Ravian L. van Ineveld, Sepide Derakhshan, Sanne de Haan, Emmy Dolman, Philip Lijnzaad, Lars Custers, Harry Begthel, Hindrik H. D. Kerstens, Lindy L. Visser, Maarten Rookmaaker, Marianne Verhaar, Godelieve A. M. Tytgat, Patrick Kemmeren, Ronald R. de Krijger, Reem Al-Saadi, Kathy Pritchard-Jones, Marcel Kool, Anne C. Rios, Marry M. van den Heuvel-Eibrink, Jan J. Molenaar, Ruben van Boxtel, Frank C. P. Holstege, Hans Clevers, Jarno Drost

**Affiliations:** 1 Oncode Institute, Princess Máxima Center for Pediatric Oncology, Heidelberglaan 25, 3584 CS Utrecht, The Netherlands; 2 Oncode Institute, Hubrecht Institute, Royal Netherlands Academy of Arts and Sciences and University Medical Center, Uppsalalaan 8, 3584 CT Utrecht, The Netherlands; 3 University Medical Center, Department of Nephrology and Hypertension, Heidelberglaan 100, 3584 CX Utrecht, The Netherlands; 4 Princess Máxima Center for Pediatric Oncology, Heidelberglaan 25, 3584 CS Utrecht, The Netherlands; 5 University Medical Center, Department of Pathology, Heidelberglaan 100, 3584 CX Utrecht, The Netherlands; 6 University College London, UCL Great Ormond Street Institute of Child Health, 30 Guilford Street, London, WC1N 1EH UK; 7 Hopp Children’s Cancer Center (KiTZ), Im Neuenheimer Feld 280, 69120 Heidelberg, Germany; 8 Division of Pediatric Neurooncology, German Cancer Research Center (DKFZ) and German Cancer Research Consortium (DKTK), Im Neuenheimer Feld 280, 69120 Heidelberg, Germany

**Keywords:** Cancer, Cancer models, Paediatric cancer, Stem cells, Adult stem cells

## Abstract

Kidney tumours are among the most common solid tumours in children, comprising distinct subtypes differing in many aspects, including cell-of-origin, genetics, and pathology. Pre-clinical cell models capturing the disease heterogeneity are currently lacking. Here, we describe the first paediatric cancer organoid biobank. It contains tumour and matching normal kidney organoids from over 50 children with different subtypes of kidney cancer, including Wilms tumours, malignant rhabdoid tumours, renal cell carcinomas, and congenital mesoblastic nephromas. Paediatric kidney tumour organoids retain key properties of native tumours, useful for revealing patient-specific drug sensitivities. Using single cell RNA-sequencing and high resolution 3D imaging, we further demonstrate that organoid cultures derived from Wilms tumours consist of multiple different cell types, including epithelial, stromal and blastemal-like cells. Our organoid biobank captures the heterogeneity of paediatric kidney tumours, providing a representative collection of well-characterised models for basic cancer research, drug-screening and personalised medicine.

## Abstract (web summary)

Pre-clinical cell culture models capturing the heterogeneity of childhood kidney tumours are limited. Here, the authors establish and characterise an organoid biobank of tumour and matched normal organoid cultures from over 50 children with different subtypes of kidney cancer.

## Introduction

Although cure rates for children with cancer have significantly increased in recent decades, cancer is still the leading cause of death by disease in the Western world among children over 1 year of age^1,2^. Renal malignancies account for ~7% of all childhood cancers and comprise multiple distinct subtypes that greatly differ in appearance and prognosis. The majority are Wilms tumours, representing ~90% of cases^3^. The most common malignant non-Wilms tumour subtypes include malignant rhabdoid tumours of the kidney (MRTK), renal cell carcinomas (RCC), clear cell sarcomas of the kidney (CCSK) and congenital mesoblastic nephromas (CMN), a rare renal neoplasm of which, in the case of stage III disease, ~25% relapse^4^. Overall survival of children with Wilms tumour has greatly improved. Yet, few effective treatment options exist for high-risk Wilms and most non-Wilms tumours^5–7^. Moreover, survivors have significant risks of late effects of the harsh treatment regimen^8^.

Wilms tumour is histologically characterised by a tri-phasic pattern, with blastemal, epithelial and stromal cell components^9^. Wilms tumour risk stratification is based on histological classification, where tumours with a high percentage of blastemal cells after pre-operative chemotherapy, or diffuse anaplastic features (hyperchromasia, atypical mitotic figures and marked nuclear enlargement) represent the high-risk group. Wilms tumours are genetically heterogeneous as well. Many different driver mutations have been described, including *WT1*, *CTNNB1, WTX*, *SIX1*, *SIX2* and microRNA-processing genes, but all with relatively low recurrence^10–13^. In addition, over 50% of Wilms tumours contain copy number alterations (CNAs)^14–17^. The non-Wilms tumour subtypes are histologically as well as genetically distinct. At least 95% of MRTKs harbour inactivating mutations in the SWI/SNF protein complex member *SMARCB1* (*SNF5*/*INI1*)^18^, whereas RCCs commonly harbour Xp11.2 or t(6;11) translocations, affecting transcription factor E3 (TFE3) and EB (TFEB), respectively^19,20^.

Pre-clinical cell culture models sustaining efficient and long-term in vitro propagation of patient-derived paediatric kidney tumour tissue have not been developed so far. Overall, in vitro cell culture models for these tumours are scarce. Cancer cell lines represent the most commonly used pre-clinical model system. Although the few available models have contributed significantly to our understanding of tumorigenesis, it has been challenging to develop cell lines capturing the phenotypic and genetic heterogeneity of paediatric kidney tumours^7^. This lack of physiologically relevant pre-clinical models for functional analyses hampers therapeutic innovation.

Three-dimensional (3D) organoid culture models open opportunities for both fundamental and translational cancer research^21^. Originally established for mouse small intestine^22^, organoids can currently be grown from primary patient material of a wide range of healthy and tumour tissues, such as colon^23^, prostate^24^, pancreas^25,26^, liver^27^, gastric^28,29^ and breast cancer^30^. Tumour-derived organoids recapitulate and maintain the genetic heterogeneity of native tumour tissue over time^25,27,30–34^, and have predictive value for individual patient drug responses^35^. Organoid technology is of particular interest for less frequently occurring cancers, such as paediatric tumours, as it allows for the generation of large collections of living material for research purposes, despite their relative rarity and small tumour sample sizes.

Here, we describe the establishment, characterisation and several applications of the first organoid biobank for paediatric cancers. It contains tumour and matching normal organoid cultures from over 50 children with renal tumours and covers a large spectrum of different subtypes, including Wilms tumours, malignant rhabdoid tumours, renal cell carcinomas and congenital mesoblastic nephromas. The malignant rhabdoid tumour organoids represent the first organoid model allowing long-term expansion of tumours of non-epithelial origin.

## Results

### A living paediatric kidney tumour organoid biobank

**Fig. 1:**
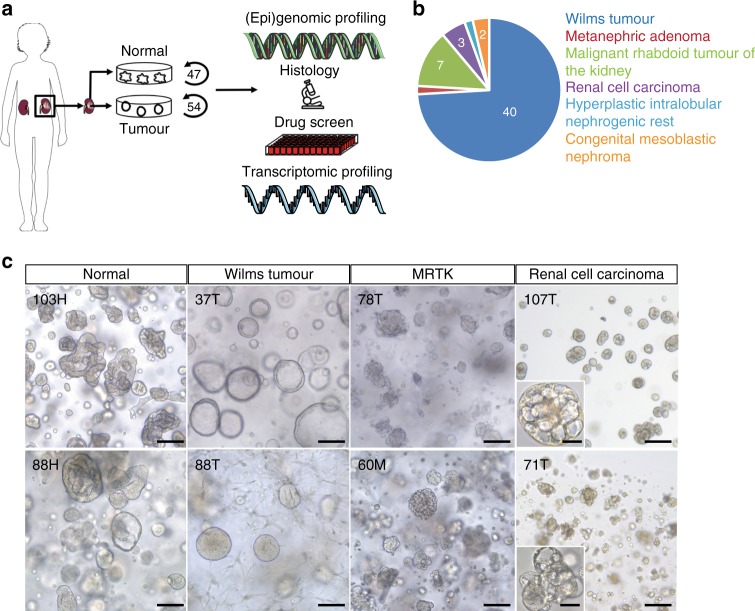
Establishment of a biobank of paediatric kidney cancer organoids. **a** Overview of the procedure to generate and characterise paediatric kidney cancer organoids. Organoids were established from tumour and, if available, matching normal kidney tissue. Organoids were subsequently characterised using histology, whole-genome sequencing (WGS), RNA sequencing (RNA-seq) and DNA methylation profiling. Lastly, drug screens were performed on a subset of Wilms tumour organoids. Modified from Servier Medical Art, licensed under a Creative Common Attribution 3.0 Generic License. **b** Pie chart representing the composition of the paediatric kidney cancer organoid biobank, consisting of organoids derived from Wilms tumours, MRTKs, RCCs, nephrogenic rests, metanephric adenoma and congenital mesoblastic nephromas. Clinical characteristics can be found in Supplementary Table 1. **c** Representative brightfield microscopy images of normal kidney tissue-derived organoids, Wilms tumour organoids, MRTK organoids and RCC organoids (*n* = 3). H healthy, T primary tumour, M metastasis. Scale bar: 100 µm, zoom in 20 µm.

We obtained tumour and matching normal kidney tissue from children that underwent nephrectomy or biopsy following informed consent (Fig. 1a; Supplementary Table 1). Tissue was minced, and cells were isolated through a combination of enzymatic digestion and mechanical disruption. A key modification of the recently published human normal kidney organoid (tubuloid) protocol^36^ was the addition of the Rho-associated coiled–coil containing protein kinase (ROCK) inhibitor Y-27632 during tissue processing. The ROCK inhibitor increases the survival of single cells in suspension by inhibition of anoikis^22,37^. Using this improved protocol, we established 54 organoid lines from different paediatric kidney tumour subtypes. These included a broad spectrum of paediatric kidney tumours, comprising Wilms tumour, MRTK, RCC, nephrogenic rest and metanephric adenoma. Of these, four known syndromal tumours (Beckwith–Wiedemann) and tumours with or without pre-surgery chemotherapy were included (Supplementary Table 1). In the majority of cases (47 out of 54), organoids were also generated from matched normal kidney tissue (Fig. 1a, b; Supplementary Table 1). Efficiency of establishment (defined as organoid growth for at least five passages) was 100% for normal tissue and 75% for Wilms tumours (40/53), 100% for MRTK (7/7) and 75% for RCC (3/4). Organoids could not always be established from chemo-treated Wilms tumour and RCC tissue due to vast amounts of necrotic tissue, whereas an efficiency of ~100% was reached from chemo-naive tissue. In addition, organoids could be established from very rare kidney tumour subtypes, including CMNs (2/2), metanephric adenoma (1/1), and from a nephrogenic rest (1/1) (Fig. 1b).

Typically, kidney tumour organoids appeared within 7 days after seeding, and can be first passaged after approximately 10–14 days. Expansion rates vary within and among different tumour types. Wilms tumour organoids can typically be passaged 1:2–1:3 every 10–14 days (>20 passages), and MRTKs weekly with 1:3 split ratios (>20 passages). Two chemo-treated RCCs were succesfully maintained for ~10 passages with 1:2–1:3 splits every 14 days, whereas chemo-naive RCC could be expanded for >20 passages.

As previously described^36^, cultures derived from normal kidney tissue consistently contained a mixture of folded and cystic organoid structures (Fig. 1c). Organoids established from tumour tissue typically displayed a different phenotype than organoids derived from the matching normal kidney tissue (Fig. 1c; Supplementary Fig. 1), giving a first indication of a tumour origin. Wilms tumour organoid lines of independent patients differed greatly in their appearance. For instance, some displayed a mix of different cell types (epithelial- and stromal-like), while others displayed a completely cystic appearance from the start. MRTK-derived organoids typically appeared in grape-like clumps of cells, while RCC organoids presented as small, cell-dense, non-cystic structures (Fig. 1c).

We next set out to analyse the organoids in-depth by means of histology, whole-genome DNA sequencing (WGS), (single cell) transcriptome analyses (RNA-seq) and DNA methylation analyses (Fig. 1a).

### Phenotypic characterisation of the kidney tumour organoids

**Fig. 2:**
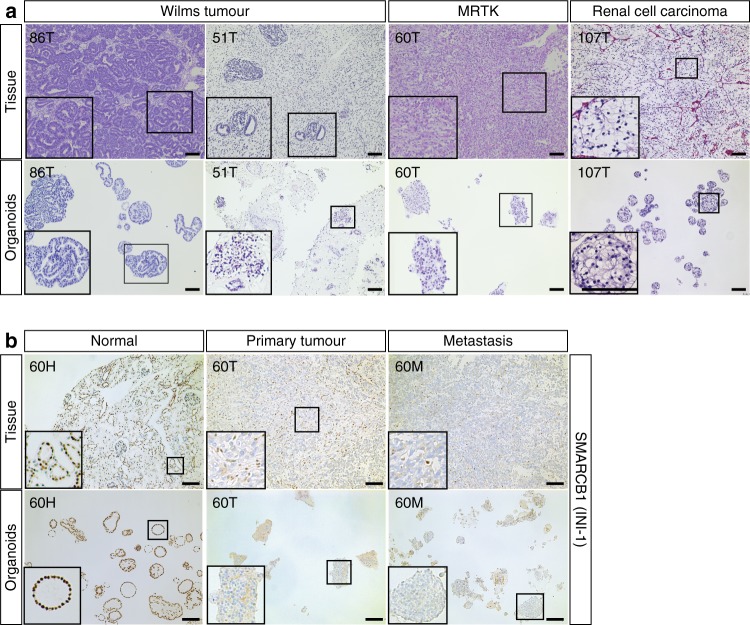
Histologic characterisation of paediatric kidney cancer organoids. **a** H&E staining on tissue (top) and matching organoids (bottom) derived from the indicated tumour types (*n* = 3). Additional cases can be found in Supplementary Fig. 2. Scale bars: 100 µm, zoom in 50 µm. **b** Representative SMARCB1 staining on normal (left), primary tumour (middle) and metastasis (right) tissue (top) and matching organoids (bottom) of a patient with a MRTK (*n* = 3). Of note, immune cells stain positive for SMARCB1 in MRTK tissue. Additional cases can be found in Supplementary Fig. 3. Scale bars: 100 µm.

**Fig. 3:**
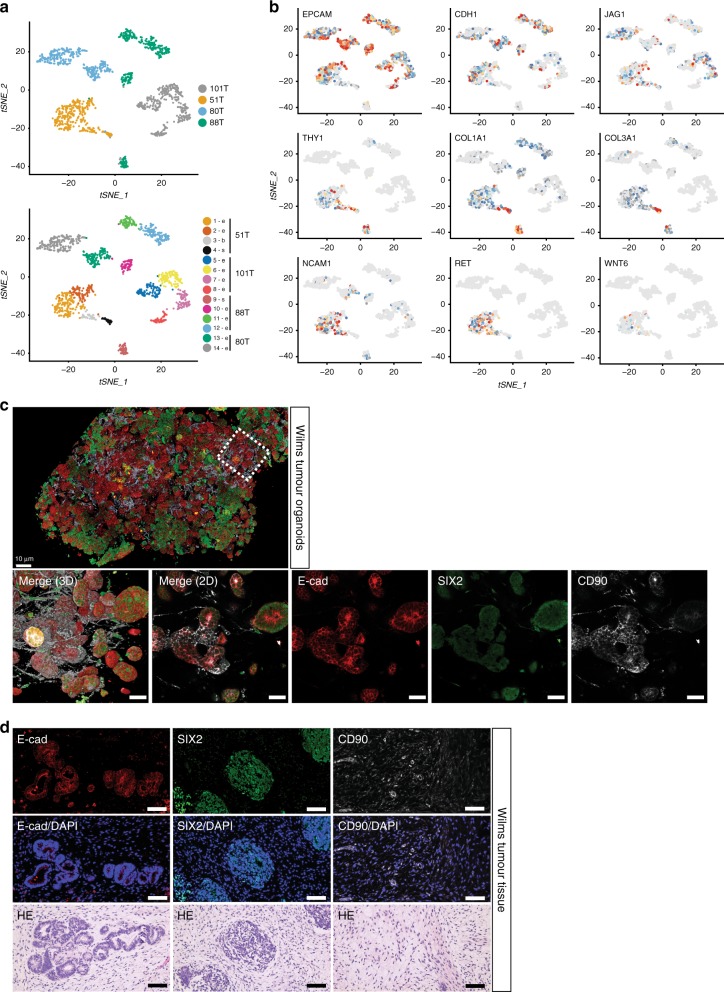
Cellular heterogeneity within Wilms tumour organoid cultures. **a** t-SNE representation of single cells from four Wilms tumour organoid lines (51T, 80T, 88T and 101T). Cells are coloured by organoid of origin (top panel) or clustering (bottom panel). Indicated are the cell types the populations are representing based on marker gene expression (see panel **b**). e: epithelial, s: stromal, b: blastemal-like. **b** t-SNE maps showing the colour-coded logged expression levels of several markers for each population demonstrating that different cell types are present in 51T and 88T, whereas 80T and 101T organoids primarily consist of different epithelial subpopulations, which is in line with their histological appearance. **c** High-resolution 3D imaging of 51T Wilms tumour organoids immunolabeled for E-cadherin (E-cad; red), SIX2 (green) and CD90 (white). Bottom panels depict enlargement from top panel in 3D (left panel) and a representative optical section (others panels). Scale bars, 100 µm (top) and 50 µm (bottom). Images are representative of *n* = 2 independent experiments. **d** Immunofluorescence imaging on 51T Wilms tumour tissue sections immunolabeled for E-cadherin (E-cad; red), SIX2 (green) and CD90 (white). Scale bars 100 µm. Images are representative of *n* = 2 independent experiments.

Current classification of the different paediatric kidney cancer subtypes is based on histological examination. To determine whether phenotypic features are retained in vitro, we histologically characterised the paediatric kidney cancer organoids. This revealed that the tumour organoids generally resembled the parental tumour tissue (Fig. 2a; Supplementary Figs. 1–3). Moreover, the tri-phasic nature (epithelium, stroma and blastema) of Wilms tumours appeared to be retained in the organoid cultures (Fig. 2a; Supplementary Fig. 2). To verify that organoid cultures contain different Wilms tumour cell types, we performed single-cell RNA-sequencing (scRNA-seq) analyses on four different Wilms tumour organoid lines; two organoid cultures with a primarily epithelial appearance (80T, 101T) and two with a mixed appearance (88T, 51T; Supplementary Fig. 4a). As expected, tumour organoid cultures primarily clustered in t-distributed stochastic neighbour embedding (t-SNE) plots based on the individual patient (Fig. 3a; Supplementary Fig. 4b). For instance, enrichment for *IGF2* and *H19* expression was detected in 51T, 80T and 88T, but was lacking in 101T (Supplementary Fig. 5a). This suggests loss of imprinting of this locus in these three lines, which is a common event in Wilms tumours^12,13,17,38^. Within organoid lines, different clusters could be distinguished as well. Whereas, 101T and 80T demonstrated a rather heterogeneous composition of different epithelial subpopulations (all marked by *EPCAM* and *CDH1* (E-cadherin) expression), distinct cell populations could be distinguished in 51T and 88T (Fig. 3a, b; Supplementary Figs. 4c–e, 5, Supplementary Data 1). Organoid culture 88T demonstrated distinct clustering of three populations. Two of these demonstrated high levels of *EPCAM* and *CDH1*, therefore likely reflecting epithelial subpopulations. The third population showed strong enrichment for stromal markers such as multiple collagens, thus representing stromal cells (Fig. 3a, b; Supplementary Figs. 4c–e and 5). In 51T, one population was enriched for epithelial markers (e.g., *EPCAM*, *CDH1*), therefore representing epithelial cells. A second population showed strong enrichment for stromal markers (e.g., collagens), whereas a third population appeared more undefined, co-expressing markers of both epithelial and stromal cells, but also more progenitor-like markers involved in neuro- and nephrogenesis (Fig. 3a, b; Supplementary Figs. 4c–e and 5). Although no exclusive markers of the blastemal compartment of Wilms tumours have been described, the latter population likely represents blastemal cells. In line with this, this population was enriched for *NCAM1* and *SIX1* expression, both proposed blastemal markers^39^. The different cell types could still be detected upon serial passaging, as determined by marker gene expression analysis using FACS and scRNA-seq on early- and late-passage cultures, although a slight enrichment was observed for epithelial progenitors (*EPCAM*, *CDH1*, *JAG1*-positive cells) and blastemal-like cells (Supplementary Figs. 5b, c and 6a). To exclude that the cultured stromal cells represent non-tumorigenic tumour-infiltrating fibroblasts, we obtained pure epithelial and stromal cells from a stromal-type Wilms tumour organoid culture (88T) based on EPCAM (epithelial) or THY1 (CD90, stromal) expression. Next, we performed targeted sequencing of bi-allelic *WT1* mutations that were identified by WGS on the bulk tumour culture (see below). Indeed *WT1* mutations could be detected in both the epithelial as well as the stromal cells, thereby confirming that the stromal cells are indeed tumour cells. Of note, matching normal kidney organoids harboured wild-type *WT1* (Supplementary Fig. 6d). Altogether, these data indicate that the cellular heterogeneity of Wilms tumours can, at least partially, be maintained in organoid cultures.

To visualise the spatial organisation of the different cell types in vitro, we performed high-resolution 3D imaging^40^ on two multi-phasic Wilms tumour organoid cultures (51T and 88T). We selected cell-type markers based on our scRNA-seq data with E-cadherin for epithelial cells, CD90 for stromal cells and SIX2 as putative blastemal marker. We observed a highly heterogeneous culture in which stromal cells formed an intricate network with epithelial organoids, as well as more blastemal-like (SIX2-positive) organoids (Fig. 3c; Supplementary Fig. 6b, Supplementary Movies 1 and 2). Similar cell types could be observed in matching Wilms tumour tissue (Fig. 3d; Supplementary Fig. 6c).

Mutations in the SWI/SNF complex member *SMARCB1* are found in >95% of rhabdoid tumours. SMARCB1 (INI-1) immunostainings are therefore routinely used to confirm MRTK diagnosis^41^. Indeed, loss of SMARCB1 expression was observed in MRTK tissue as well as in all organoids established from it, whereas strong nuclear expression was observed in normal kidney tissue from the same patient and organoids derived thereof (Fig. 2b). In some cases, a mix of grape-like clumps of cells and more cystic organoid structures was observed, pointing towards contamination of the tumour organoid culture with organoids derived from normal kidney epithelium, which was confirmed by staining for SMARCB1 (Supplementary Fig. 3b). In contrast to normal kidney tissue, MRTKs do not show epithelial differentiation^42^. Therefore, we separated MRTK cells from normal kidney cells based on expression of the epithelial marker EPCAM. As expected, no EPCAM-positive cells could be detected in MRTK organoids derived from a lymph node metastasis (Supplementary Fig. 3c). In contrast, an EPCAM-positive cell population was observed in primary tumour-derived MRTK organoids. Indeed, a pure MRTK organoid culture, devoid of epithelial normal kidney organoid structures, could be established (Supplementary Fig. 3d).

Lastly, RCC organoids consist of cells with typical clear cytoplasms (Fig. 2a; Supplementary Fig. 2), whereas tumour origin of a *TP53*-mutated RCC-derived organoid was confirmed by immunostaining for P53 (Supplementary Fig. 3e).

### Genetic characterisation of kidney cancer organoids

**Fig. 4:**
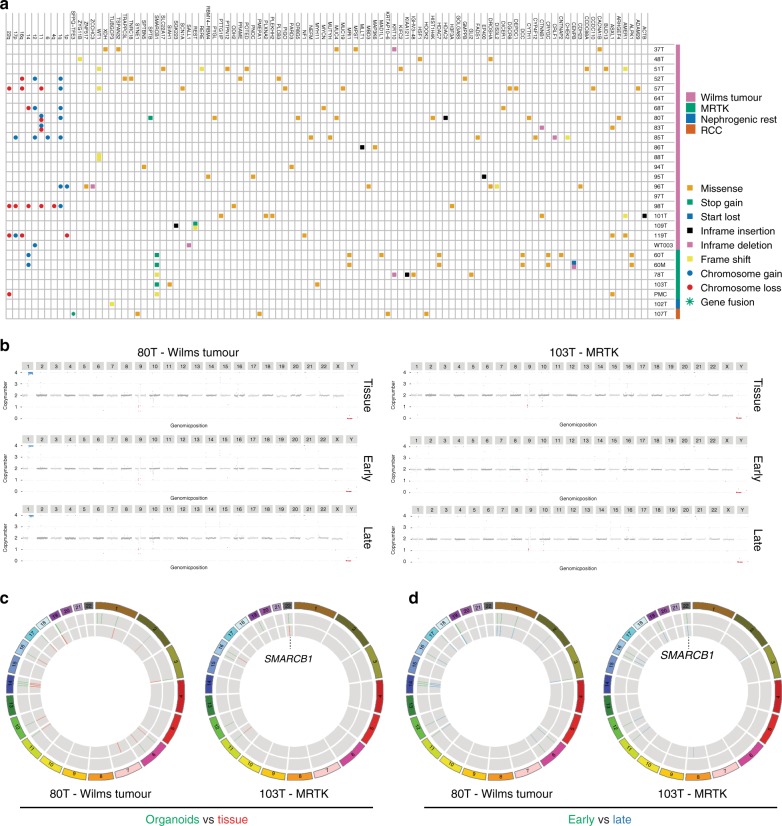
Genetic characterisation of paediatric kidney cancer organoids reveals common driver mutations and copy number alterations. **a** Overview of somatic mutations identified in paediatric kidney cancer organoids compared with their matching normal kidney organoids. When matching normal kidney organoids were not available, somatic mutations in known driver genes are indicated. Variant allele frequencies are given in Supplementary Data 2. **b** Genome-wide CNAs (karyograms) and coding gene mutations (circos plots) (**c**, **d**) in matching tumour tissue vs organoid and early vs serially passaged (P4, P5 vs P10, P11) reflecting ~3 months of culturing) organoid pairs reveal that organoids recapitulate the genetic landscape of the tissue they were derived from and that this genetic landscape is retained over time.

Several recent studies have revealed the heterogeneous genetic landscape of Wilms tumours^12,13,17^. In addition to a significant percentage of chromosomal alterations, numerous mutated genes have been described, although all with relatively low frequency. To characterise the mutations and CNAs in kidney tumour organoids, we performed whole-genome sequencing (WGS) on 28 tumour organoids and compared these, when available, to their matching normal organoid counterparts. Nine out of 20 Wilms tumour organoids showed CNAs such as gain of 1q, 6, 12 and 17p, and loss of 1p, 4q, 16q, 17p, 14, 11 and 22 (Fig. 4a; Supplementary Fig. 7a), which is consistent with previous reports^12,13,17,43^. Moreover, mutations in typical Wilms tumour genes were identified, such as *WT1*, *DIS3L2*, *WTX*, *CTNNB1* and the miRNA-processing genes *DROSHA*, *DGCR8* and *DICER1* (Fig. 4a; Supplementary Data 2). In a few cases, no common tumour mutations could be detected. We detected a fusion of the *TFE3* gene with the *SFPQ* gene in an RCC-derived organoid culture (107T, Fig. 4a), a frequently occuring event in paediatric RCCs^44^. In MRTK organoids, *SMARCB1* was the only recurrent mutated gene, varying from nonsense mutations to losses of chromosome 22q, on which locus *SMARCB1* is encoded. As previously described^45^, no apparent differences in the total number of somatic mutations were found between Wilms tumours and MRTKs (Supplementary Fig. 7b, d). Moreover, we detected a heterozygous *KRAS*^G12R^ mutation in metanephric adenoma tissue as well as in the organoids derived thereof, whereas this mutation was absent in matching normal kidney tissue and organoids (Supplementary Fig. 7c). To determine whether paediatric kidney cancer organoids genetically recapitulate the tumours from which they were derived, we performed WGS on five tumour organoids and matching tumour tissues (three Wilms tumours, two MRTKs). Indeed, this demonstrated that organoids are genetically highly similar to their tumour tissue counterparts (Fig. 4b, c; Supplementary Fig. 8). Lastly, genetic characterisation of early and serially passaged tumour organoids confirmed their genetic stability over time (Fig. 4b, d).

To further confirm that organoids preserve the genetic landscape of native tumours, we extracted mutational signatures^46,47^ from the WGS data of our organoids, as well as from recently published WGS data sets of Wilms tumour and brain rhabdoid tumour (ATRT) tissue^45,48^. We subsequently compared these to recently described mutational signatures in the Catalogue of Somatic Mutations in Cancer (COSMIC) database^49^ and signatures reported as paediatric cancer-specific^48^. This analysis revealed the presence of a large number of different signatures in Wilms and rhabdoid tumours, with most common occurrence of signatures 1, 5 and T10 (Supplementary Fig. 7d). No apparent therapy-related signatures were observed in the organoids derived from pre-treated tumours. Importantly, the broad spectrum of mutational signatures identified in these tumour tissues were represented in our paediatric kidney cancer organoids (Supplementary Fig. 7d).

In summary, we show that paediatric kidney cancer organoids recapitulate the diverse genomic landscape of paediatric renal tumours, such as CNAs, cancer gene mutations, as well as mutational signatures.

### Gene expression and DNA methylation profiling

**Fig. 5:**
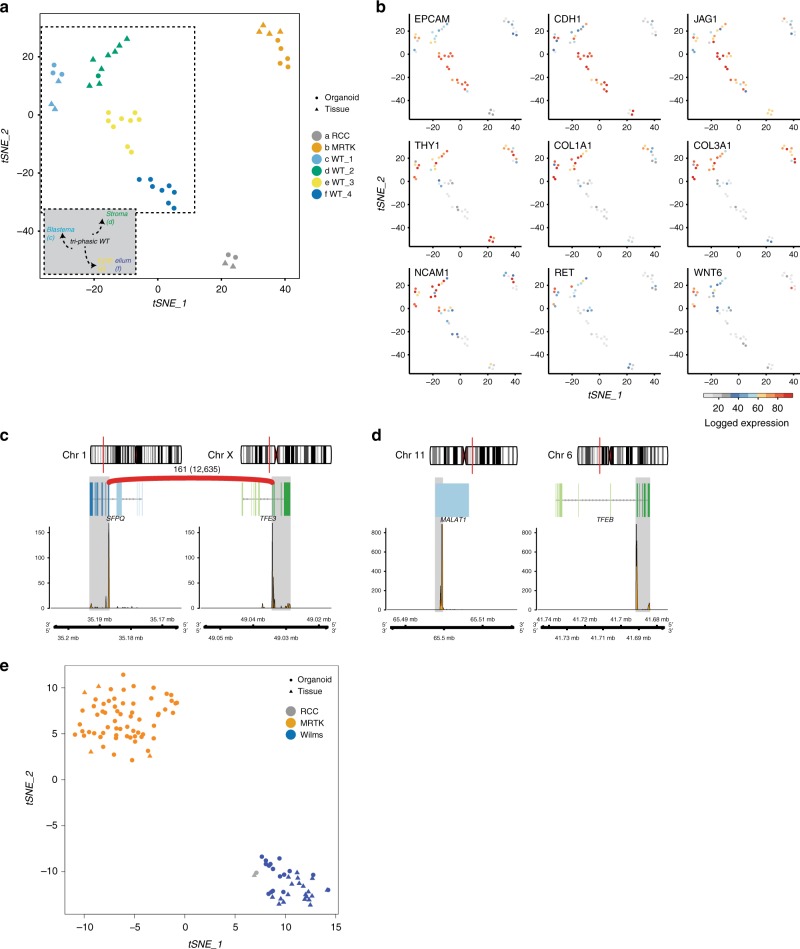
Transcriptome and DNA methylation profiling of paediatric kidney cancer organoids. **a** t-SNE representation of unsupervised graph-based clustering of paediatric kidney cancer organoids and tissues gene expression profiles, demonstrating a disease-based separation for the three main tumour types (RCC, MRTK and Wilms tumour) and a composition-based separation for the most prevalent one, Wilms tumour. **b** t-SNE maps, as in **a**, showing the colour-coded logged expression levels of several markers used in the clinic or separating the different populations. **c**, **d** Depicted are fusion transcripts detected in tRCC-derived organoids 107T (**c**) and 71T (**d**) with their chromosomal location and exon structure and a schematic representation of the fusion breakpoint. Coverage track of the fusion genes is included at the bottom, indicating RNA expression levels. The number above the red arc represents the sequencing reads that support the fusion event. **e** t-SNE analysis was performed using the top 2000 most variably methylated CpG sites in paediatric kidney cancer organoids and tissues, and revealed that organoids cluster with the tumour entity they were derived from.

To determine whether organoids represent gene expression profiles of the different paediatric kidney tumour subtypes, we performed paired-end RNA-seq on organoids derived from 29 paediatric renal tumours. In 18 cases, matching tumour tissue was available and included in the analyses. The most variable genes were used to project the RNA samples in a linear dimensional reduction space using principal components (PCs). As expected, the first PC separates the growth microenvironment of the tumour cells (organism versus in vitro), the second PC the MRTKs, while the third separates the RCCs (Supplementary Fig. 9a, b). After regressing out the growth microenvironment, the samples were clustered in sample populations (Fig. 5a). The resulting clusters separate the samples based on their tumour diagnosis. The first two sample clusters comprise the RCC and MRTK tissue and organoid samples (a and b, respectively; Fig. 5a), demonstrating that RCC and MRTK organoids retain the identity of the native tumour tissue. Wilms tumour tissue and organoid samples were more heterogeneous separating into the remaining four clusters (Fig. 5a). Cluster c has high levels of progenitor-like blastema markers. The clustering of 51T sample in cluster c thus supersedes the expression of epithelial markers *EPCAM* and E-cadherin (*CDH1*) coming from the epithelial population of the 51T organoid cells (Fig. 3a). Cluster d has higher stromal expression, shown with the elevated levels of various collagen genes, while clusters e and f have progressively higher epithelial characteristics, evident by the increased levels of *CDH1* and *EPCAM* (Fig. 5b).

Bulk gene expression profiling can also point to the presence of mechanisms of carcinogenesis, not detected by DNA sequencing. For instance, elevated expression of insulin-like growth factor 2 (*IGF2*) is reported in the majority of Wilms tumours^12,13,38,50^. In line with this, the majority of Wilms tumour organoids demonstrated high *IGF2* gene expression compared with normal kidney, MRTK and RCC organoids (Supplementary Fig. 10a). Wilms tumour organoids largely retained the high *IGF2* expression detected in the parental tumour tissue (Supplementary Fig. 10b). Compared with the other Wilms tumour organoid lines and normal kidney-derived organoids, WT003T showed markedly reduced *DICER1* gene expression (Supplementary Fig. 10c). WT003T was derived from a cystic partially differentiated Wilms tumour, a rare Wilms tumour subtype composed of large cysts separated by septa. Remarkably, cystic nephroma, another childhood renal tumour composed of large cysts, has been previously linked to *DICER1* mutations^51–54^. In contrast to MRTKs, no significant *hTERT* expression could be detected in Wilms tumour tissue and organoids derived thereof (Supplementary Fig. 10d), which is in contrast to a study by Dome et al.^55^ demonstrating *hTERT* expression in Wilms tumours using quantitative RT-PCR. Still, the vast majority of Wilms tumour organoid cultures could be long-term propagated, suggesting that alternative mechanisms are involved to maintain replicative potential, as previously suggested^56^.

The RNA-seq analyses demonstrated expression of the *SFPQ-TFE3* fusion transcript in 107T RCC organoids (Fig. 5c; Supplementary Fig. 10e), thus confirming the WGS analyses (Fig. 4a). In a second RCC-derived organoid line (71T), we detected a *MALAT1-TFEB* fusion transcript (Fig. 5d; Supplementary Fig. 10f). Both these fusions have been described as drivers in paediatric RCCs^44^. Strikingly, we detected strongly decreased *TP53* transcript levels in 71T and 107T organoids as well as tissue (Supplementary Fig. 9c), which confirms previous reports^57^. Thus, tumour organoids display representative gene expression profiles, which allow unsupervised separation of the majority of paediatric kidney cancer subtypes.

Finally, we set out to determine whether paediatric kidney cancer organoids retain the epigenetic profile of their corresponding tumour entity. We therefore performed DNA methylation analyses on a subset of tumour organoids and compared these to the DNA methylation profiles of matching tumour tissues as well as recent DNA methylation data of malignant rhabdoid tumours^58^. In line with the transcriptome analyses, clustering analysis demonstrated that organoids clustered with their respective tumour types and thus maintained the epigenetic profile of the tumour (Fig. 5e), as found previously for colorectal cancer organoids^59^.

### Gene editing and high-throughput drug screens

*TP53* mutations positively correlate with anaplasia^60–62^. To determine whether paediatric kidney tumour organoids can be genetically manipulated, we set out to model anaplastic Wilms tumours by generating *TP53*-knockout mutations in *TP53*-wild-type Wilms tumour organoids using CRISPR/Cas9 gene editing. We transiently transfected the 80T Wilms tumour organoid culture with either a control or *TP53* targeting sgRNA. Three days after transfection, we added nutlin-3 to the medium to select for *TP53*-mutant organoids (Supplementary Fig. 11a)^63^. As expected, control sgRNA-transfected Wilms tumour organoids died upon nutlin-3 treatment (Supplementary Fig. 11b). Surviving organoids in the *TP53* sgRNA-transfected culture were clonally expanded, and homozygous *TP53* knockout was verified by genotyping and western blot (Supplementary Fig. 11c, d). Subsequent histological characterisation did not reveal any distinct anaplastic features in *TP53*-knockout Wilms tumour organoids (Supplementary Fig. 11e). This suggests that loss of *TP53* is required but not sufficient for inducing anaplasia in Wilms tumours, which is in line with the findings of Wegert et al.^62^ describing *TP53* mutations in regions lacking signs of anaplasia. Possibly, persistent chromosome instability caused by the loss of *TP53* is required for the acquisition of an anaplastic phenotype.

**Fig. 6:**
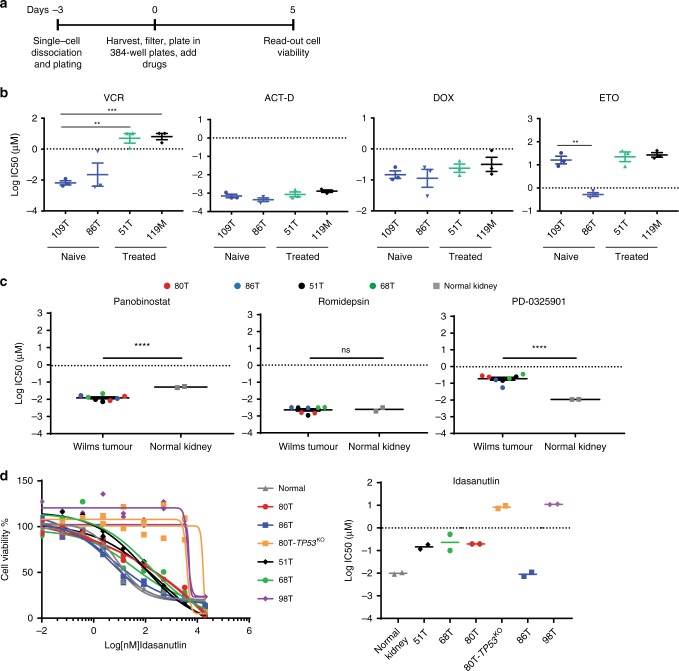
Organoid drug screens reveal patient-specific drug sensitivities. **a** Schematic overview of the organoid drug treatment experiment. **b** Graphs show the average IC_50_ values of vincristine (VCR), actinomycin D (ACT-D), doxorubicin (DOX) and etoposide (ETO) in the indicated Wilms tumour organoid lines. In case the IC_50_ value was not reached (see Supplementary Fig. 12a), the highest tested concentration was used for the calculations. Error bars represent SEM of three independent experiments (each individual experiment includes technical quadruplicates). *P*-values were calculated using a two-tailed Student’s *t* test, two-sided: **<0.01, ***<0.001. *P*-value VCR: 119 M vs 109T = 0.0005; 109T vs 51T = 0.0049. *P*-value ETO: 109T vs 86T = 0.0044. **c** Average IC_50_ values of romidepsin, panobinostat and PD0325901 in the indicated Wilms tumour and normal kidney organoid cultures. Each dot/square (two per organoid culture) represents the average of technical quadruplicates of an individual organoid culture. *P*-values were calculated using a two-tailed Student’s *t* test, two-sided: ****<0.0001. *P*-value Romidepsin: Wilms tumour vs normal kidney = 0.8339. **d** Dose–response curves (left) and average IC_50_ (right) of Idasanutlin on the indicated Wilms tumour and normal kidney organoid cultures. As control for P53 function, 80T-*TP53*^KO^ organoids were included, thereby demonstrating that anaplastic Wilms tumour-derived organoids (98T) are less sensitive to Idasanutlin treatment. Curves with the same colour represent independent experiments. Each individual point represents the average of quadruplicate measurements. Source data are provided as a Source Data file.

In order to determine whether our patient-derived Wilms tumour organoids can be used as a drug-screening platform, we first tested their sensitivity towards standard-of-care chemotherapeutics. The current chemotherapy regimen encompasses actinomycin D (ACT-D) and vincristine (VCR) prior to radical nephrectomy, possibly followed by doxorubicin (DOX) and/or etoposide (ETO)^5^. We used two chemo-naive (109T, 86T) Wilms tumour organoid lines and two that were derived of chemo-treated Wilms tumours (51T, 119M) and subjected those to a previously established drug-screening platform and cell viability read-out^33^ (Fig. 6a). Testing six different concentrations per drug allowed us to generate reproducible dose–response curves (Supplementary Fig. 12a) and calculate half-maximal inhibitory concentrations (IC_50_) (Fig. 6b). While organoids derived from both pre-treated Wilms tumours were significantly less sensitive to VCR than the untreated Wilms tumour organoids, similar sensitivity was observed towards ACT-D. This suggests that remaining viable Wilms tumour cells after pre-operative chemotherapy are more resistant to VCR and demonstrates the added value of the combination treatment. Interestingly, organoid line 86T demonstrated markedly higher sensitivity to etoposide compared with all other lines. Yet, no apparent genetic biomarker could be identified in our WGS data. Next, we screened four Wilms tumour organoid lines using a ~150 compound library with six different concentrations (Supplementary Table 2). Ranking compounds based on the calculated area under the estimated dose–response curve (AUC) revealed multiple different MEK and HDAC inhibitors in the top 25 of most effective compounds (Supplementary Fig. 12b). We then validated the most potent MEK and HDAC inhibitors (Romidepsin, Panobinostat and PD0325901) and additionally included normal kidney organoids to determine tumour-specific targeting. This showed that normal kidney organoids are equally sensitive to Romidepsin (HDAC 1/2 inhibitor) and even more sensitive to MEK inhibition (Fig. 6c; Supplementary Fig. 12c). Interestingly, Wilms tumour organoids demonstrated significantly increased sensitivity to Panobinostat (pan-HDAC inhibitor) compared with normal kidney organoids (Fig. 6c; Supplementary Fig. 12c), thereby possibly pointing towards a less toxic therapeutic strategy. Lastly, we established organoids from an anaplastic Wilms tumour (98T), which are characterised by mutations in *TP53*^60–62^. Indeed, 98T organoids expressed reduced *TP53* transcript levels (Supplementary Fig. 9c) and showed several anaplastic characteristics (enlarged nuclei and hyperchomasia, Supplementary Fig. 12d). To test for P53 functionality, we next tested 98T organoids together with the other (non-anaplastic) Wilms tumour-derived organoid lines for sensitivity to Idasanutlin, a therapeutic P53 stabilising agent. In addition, we included our genetically engineered 80T-*TP53*^KO^ organoids as a control. A dramatically reduced sensitivity for Idasanutlin was observed in 98T organoids compared with all other Wilms tumour organoids (Fig. 6d). The observed sensitivity was comparable with the sensitivity of 80T-*TP53*^KO^ organoids, indicating that P53 function is severely hampered in these organoids. Of note, as for etoposide, 86T organoids demonstrated a high sensitivity for Idasanutlin (Fig. 6b, d), indicating that this tumour is particularly sensitive to P53-activating agents.

Altogether, our paediatric kidney cancer-derived organoid models are amenable to gene editing and allow for high-throughput drug testing to reveal patient-specific drug sensitivities and to make drug/mutation correlations.

## Discussion

The development of 3D cell culture systems allows for the highly efficient establishment of pre-clinical cancer models from patient-derived tissue. Availability of such models is of particular interest for less common cancers, such as paediatric tumours, as it allows for the generation of large collections of living material for research purposes, despite their relative rarity. As for adult cancers, cell lines have been the gold standard for paediatric cancer research. Yet, it has been challenging to develop cell lines capturing the phenotypic and genetic heterogeneity of paediatric kidney tumours^7^. The establishment efficiency of cancer cell lines is very low, which makes them unsuitable for the generation of patient-specific models and individualised drug screening. In the rare cases a cell line can be established from primary tumour tissue, this usually involves extensive adaptation and selection to in vitro 2D culture conditions. As only rare clones can be expanded, the derived cell lines typically undergo substantial genetic changes, and no longer recapitulate the genetic background and genetic (as well as cellular) heterogeneity of the original tumours. Wilms tumour is the most common and extensively studied paediatric renal tumour. Although Wilms tumour cell lines have contributed tremendously to our understanding of Wilms tumour biology, they almost invariably derive from advanced cancers. Moreover, the current cell line panel does not reflect the heterogeneous nature of Wilms tumours^7,64,65^. Wegert et al.^56^ recently developed a protocol for the establishment of cultures from primary Wilms tumour tissue. This protocol allowed them to generate cell cultures from 22% (12 out of 55) of Wilms tumour patients (versus 75% using our protocol). No confirmed matching healthy reference cultures were established. Moreover, these 2D monolayer cultures phenotypically did not resemble parental tumour tissue very well, with mainly fibroblast-like features and no blastemal marker expression^56^. Finally, pre-clinical in vitro cell models are scarce for the other paediatric kidney cancer subtypes. Here, we describe the establishment of the first paediatric cancer organoid biobank, containing the majority of childhood kidney cancer subtypes: Wilms tumours, MRTKs, RCCs and several rarer renal tumour entities, such as CMNs and metanephric adenomas. To our knowledge, the malignant rhabdoid tumour organoids represent the first organoid model sustaining long-term growth of tumours of non-epithelial origin. So far, we have not established organoids from CCSK tissue, due to their low occurrence. We demonstrate that epithelial, stromal and blastemal-like populations can be maintained in Wilms tumour organoids, which are organised in an intricate 3D network. Moreover, we show that paediatric kidney cancer organoids retain phenotypic, genetic, epigenetic and gene expression characteristics of native tumours to a large extent.

Besides tumour cells, clinical tumour samples typically contain areas of necrotic tissue as well as non-tumour cells (e.g., blood vessels, immune and stromal cells). This low tumour content of clinical samples can severely hamper molecular characterisation and drug-sensitivity readouts. Tumour organoids are pure tumour cultures, therefore allowing for in-depth characterisation of tumour cell-specific features and drug sensitivities. Moreover, the possibility to propagate and expand primary tumour tissue as organoids provides nearly limitless availability of material for research purposes. Importantly, we and others have demonstrated that tumour organoids largely retain the heterogeneity of the tissue they were derived from over serial passaging^27,34,66,67^. Since clonal dynamics within tumour organoid cultures may occur^34^, early passage organoid cultures should preferentially be used for therapy development purposes. Similarly, variability induced by organoid production procedures might influence experimental readouts. Comprehensive genetic and phenotypic analyses of the cultures should therefore be considered before their utilisation in downstream experimental procedures. The lack of blood vessels and immune cells are an intrinsic limitation of organoid technology as well. Recent efforts led to the development of co-cultures of tumour organoids with cancer-associated fibroblasts^68,69^ and immune cells^70^, demonstrating the potential of implementing these factors in paediatric kidney organoid cultures. Here, we report that organoids can be derived with high efficiency from the majority of paediatric kidney tumours and can be rapidly expanded, which allows for identifying patient-specific drug sensitivities, and potentially the development of improved therapeutic strategies.

## Methods

### Human tissue

All experiments with human tissue were approved by the medical ethical committee of the Erasmus Medical Center (Rotterdam, the Netherlands). The parents of all patients participating in the biobank study signed informed consent forms approved by the responsible authority.

### Tissue processing

Following nephrectomy or biopsy, a random piece was selected from viable tumour tissue and, when available, normal kidney tissue. One or two random pieces were fixed in formalin for histopathological analysis. The remainder was minced into ~1-mm^3^ pieces. Several pieces were snap frozen and stored at −80 °C for DNA and RNA isolation. The remainder was digested in AdDF+++ (Advanced DMEM/F12 containing 1× Glutamax, 10 mM HEPES and antibiotics) containing 1 mg ml^−1^ collagenase (Sigma, C9407) and 10 µM Y-27632 on an orbital shaker for 45 min at 37 °C. Next, the suspension was washed with AdDF+++ followed by centrifugation at 250×*g*. In case of a visible red pellet, erythrocytes were lysed in 1–2 mL red blood cell lysis buffer (Roche, 11814389001) for 5 min at room temperature before the addition of 10 ml AdDF+++ and centrifugation at 250×*g*.

### Organoid culture

The cell pellets were seeded in growth factor-reduced BME (Trevigen, 3533-010-02) and cultured in kidney organoid medium (AdDF+++ supplemented with 1.5% B27 supplement (Gibco), 10% R-spondin-conditioned medium, EGF (50 ng ml^−1^, Peprotech), FGF-10 (100 ng ml^−1^, Peprotech), N-acetylcysteine (1.25 mM, Sigma), Rho-kinase inhibitor Y-27632 (10 µM, Abmole) and A83-01 (5 µM, Tocris Bioscience)^36^. Medium was changed every 3–4 days, and organoids were passaged every 1–3 weeks. Depending on organoid morphology, organoids were either passaged using mechanical dissociation (Wilms tumour organoids, MRTK organoids), or TrypLE Express (Invitrogen, 12605036) containing 10 µM Y-27632 (Wilms tumour organoids, RCCs). Following the addition of 5–10 ml AdDF+++ and centrifugation at 250×*g*, cells were reseeded in BME and topped with kidney organoid medium. All organoid cultures are stored in the biobank of the Princess Máxima Center and made available to the scientific community according to the rules and regulations under which the patients and parents gave informed consent for donating the tissue.

### Organoid transfection and genotyping

Organoids were transfected using lipofection as previously described^63^. In brief: organoids were digested to single-cell suspensions using TrypLE Express with 10 µM Y-27632. Cells were subsequently resuspended in 450 µl kidney organoid medium and plated in 48-well plates at high density (80–90% confluent). Nucleic acid–Lipofectamine 2000 complexes were prepared according to the standard Lipofectamine 2000 protocol (Invitrogen). Four microlitres of Lipofectamine 2000 reagent in 50 µl Opti-MEM medium (Gibco) and 1.5 µg of DNA (pSpCas9(BB)-2A-GFP control or sgRNA *TP53* plasmid in 50 µl Opti-MEM medium) were mixed together, incubated for 5 min and added to the cells. The plate was centrifuged at 600 *g* at 32 °C for 1 h, and incubated for 4 h at 37 °C before single cells were plated in BME. Three days after transfection, 10 µM nutlin-3 (Cayman Chemical) was added to the growth medium. After approximately 2–3 weeks, surviving clones were picked and clonally expanded. For genotyping, genomic DNA was isolated using Viagen Direct PCR (Viagen). Primers for the PCR amplification using GoTaq Flexi DNA polymerase (Promega) were as follows:

*TP53*_for 5′-TGGACCCTCTGAACTGCAGCAT-3′; *TP53*_rev 5′-CAGGAAGCCAAAGGGTGAAGA-3′.

For *WT1* genotyping, DNA was isolated from FACS purified EPCAM+/CD90− and EPCAM−/CD90+ cells using Viagen Direct PCR (Viagen). Primers for the PCR amplification using GoTaq Flexi DNA polymerase (Promega) were as follows: *WT1*_exon10_for 5′-TTTCCAGAAGCACCGGTATC-3′; *WT1*_exon10_rev 5′-TGGCCAAGTTGTCAGAAAAA-3′; *WT1*_exon7_for 5′-TTATTGCAGCCTGGGTAAGC-3′; *WT1*_exon7_rev 5′-GGAGTGTGAATGGGAGTGGT-3′. Products were cloned into pGEM-T Easy vector system I (Promega) and subsequently sequenced using T7 sequencing primer.

### Histology, immunohistochemistry and immunofluorecence

Tissues and organoids were fixed in 4% paraformaldehyde, dehydrated and embedded in paraffin. Immunohistochemistry was performed according to standard protocols on 3–4 µm sections. Sections were subjected to H&E, immunohistochemical as well as immunofluorescence staining. The following primary antibodies were used for immunohistochemical staining: desmin (Leica Novacastra, NCL-L-Des-Der11, 1:100), INI-1 (BD Transduction Laboratories, 612111, 1:400), P53 (Dako, M7001, 1:6000). For immunofluorescence on tissues: SIX2 (Proteintech, 11562-1-AP, 1:200), E-cadherin clone ECCD-2 (ThermoFisher, 13-1900, 1:200), CD90 clone EPR3133 (Abcam, 133350, 1:100) were used. Imaging was performed using Leica DM6 microscope.

### High-resolution 3D organoid imaging

High-resolution 3D imaging on organoids was performed as described^40^ using the following antibodies: SIX2 (Proteintech, 11562-1-AP, 1:200), E-cadherin clone ECCD-2 (ThermoFisher, 13-1900, 1:500), CD90-APC clone 5E10 (BioLegend, 328113, 1:200). Imaging was performed using Zeiss LSM880 microscope. Three-dimensional reconstruction was performed using the software Imaris v.9.2.1.

### FACS

Organoids were dissociated into single-cell suspensions using TrypLE Express (ThermoFisher) supplemented with Rho-kinase inhibitor Y-27632 (10 µM, Abmole). Single-cell suspensions were stained using mouse Alexa-fluor 488 anti-human CD326 EPCAM clone 9C4 (BioLegend, 324210, 1:20), CD90-APC clone 5E10 (BioLegend, 328113, 1:50) as described^71^. Populations were sorted using BD FACSAria—Fusion sorter (BD Biosciences) or MoFlow® Astrios (Beckman Coulter) and used for their respective applications (Supplementray Fig. 13). Data were analysed with software Kaluza analysis v2.1.

### Whole-genome sequencing and DNA methylation profiling

Genomic DNA from tissue and organoids was extracted using the ReliaPrep^TM^ gDNA Tissue Miniprep System (Promega) according to the manufacturer’s instructions and sent for WGS. Samples were sequenced on BGI-SEQ500 platform (BGI Hong Kong) or with Illumina NovaSeq6000 sequencers (Hartwig Medical Foundation) to 30× base coverage.

Sequence reads were mapped against human reference genome GRCh37 by using Burrows-Wheeler Aligner v0.7.5a mapping tool^72^ with settings “bwa mem -c 100 -M”. Sequence reads were marked for duplicates by using Sambamba v0.6.8 and realigned per donor by using Genome Analysis Toolkit (GATK) IndelRealigner v3.4-46. Full pipeline description and settings also available at https://github.com/UMCUGenetics/IAP. Mutations were called and filtered as described^73^. Briefly, raw variants were multisample-called by using the GATK HaplotypeCaller v3.4-46^74^. To obtain high-quality somatic mutation catalogs, we filtered out variants with evidence in their corresponding normal samples, overlaps with the Single Nucleotide Polymorphism Database v137.b37, and the variants that did not reach our quality measurements (base coverage of 10×, variant allele frequency (VAF) of 0.1, GATK phred-scaled quality score of 100 for base substitutions, 250 for indels and mapping quality (MQ) of 60 for indels). Indels that were present within 100 bp of a called variant in the control were excluded. For signature analysis, additional filter on GATK genotype quality (GQ) of 10 in normals, 99 in samples was applied to obtain high-quality base substitutions. For samples without matching normals, base coverage of 20× was used instead of 10×, and these were not included in signature analysis. Only autosomal variants were considered. The scripts used for the filterings are available at https://github.com/UMCUGenetics/SNVFI and https://github.com/ToolsVanBox/INDELFI. Non-synonymous mutations (missense mutation, start loss, stop gain, inframe insertion/deletion and frame shift) in all genes identified in samples with matching normals, and in known driver genes from samples without matching normals were reported as driver mutations. Every coding mutation including drivers has been manually inspected to exclude false calls. Average chromosome gain or loss were calculated based on the estimated copy number by freec^75^ using copy number package in R^76^; low copy number changes indicate partial gain or loss. Signature analysis was performed together with published Wilms tumour^48^ and atypical teratoid rhabdoid tumours^45^ using an in-house developed R package (MutationalPatterns)^77^. All single base substitution signatures that are reported as plausible in the COSMIC SigProfiler signatures (https://www.synapse.org/#!Synapse:syn11967914), except for signature 40, which is similar to signature 5 and therefore challenging to distinguish from signature 5 with a small sample set, and paediatric data specific signatures (T10 and T11^48^) were used in this analysis. Since signature 1 and 5 are associated with age, these signatures were assumed to be present in all samples. Thus, the mutational profile of every sample was re-fitted to signature 1 and 5, calculated its cosine similarity and then a signature that increases the cosine similarity the most was selected by adding and re-calculating the cosine similarity for the rest of signatures one by one. Until the overall cosine similarity reaches to 0.9 or the increase of cosine similarity by adding another signature dropped to <0.01, we repeated to add a signature with the highest increase in the cosine similarity. The total number of base substitutions and the absolute contributions of the selected signatures for each sample are reported. The script is available at https://github.com/ToolsVanBox/MutSigPipe.

DNA methylation profiles were assessed using Illumina Human MethylationEPIC BeadChip arrays at the German Cancer Research Center (DKFZ) Genomics and Proteomics Core Facility according to the manufacturer’s instructions. Analysis was performed as described^78^.

### Single-cell RNA sequencing

Samples were prepared according to the Sort-seq method^79^. In brief, organoids were dissociated into single-cell suspensions using TrypLE Express (ThermoFisher) supplemented with Rho-kinase inhibitor Y-27632 (10 µM, Abmole). Viable single cells were sorted based on forward/side scatter properties and DAPI staining using FACS (FACSJazz, BD Biosciences) into 384-well plates (Biorad) containing 10 µl mineral oil (Sigma) and 50 nl of RT primers. Samples were subsequently processed into Illumina sequencing libraries as described^79^. Libraries were sequenced paired-end at 75 bp read length using the Illumina NextSeq sequencer. Sequencing data were processed using the Sharq pipeline as described^80^. We performed the mapping using STAR version 2.6.1 on the hg38 Patch 10 human genome and read assignment with featureCounts version 1.5.2 using a gene annotation based on GENCODE version 26. Transcripts mapping to the mitochondrial genome were removed and the percentage of mitochondrial transcripts calculated. Cells with a percentage exceeding 40% of the total were excluded. In addition, cells with <1000 unique transcripts were also excluded. Genes with low expression (defined as either having less than five cells expressing the gene or less than two cells with less than two transcripts) were removed. All subsequent analyses were performed using the R package Seurat (version 3.0.2)^81^. Data were processed into a Seurat object and log normalised to 10,000 transcripts. In order to avoid the influence of specific cell processes on the clustering and visualisation, the most variable genes were filtered to remove mitochondrial pseudogenes and cell cycle effects. Genes involved in cell cycle were derived as follows: a set of well-known cell cycle markers^82,83^ were directly removed from variable genes. In addition, genes that correlate with the cell cycle process were identified. To do this, we used a set of well-known cell cycle markers^82^ to calculate S and G2M scores using Seurat’s “CellCycleScoring” function. We then correlated all genes with these two scores across all cells, obtaining per-gene correlations with S and G2M score. Using the distribution of correlations of well-known S and G2M genes with either S or G2M score, we calculated cut-offs. The S cut-off is calculated as follows:

$${\mathrm{Max}}\left( {{\mathrm{Med}}\left( {{\mathrm{S}}_{\mathrm{S}}} \right) - {\mathrm{Med}}\left( {{\mathrm{S}}_{{\mathrm{G2M}}}} \right),{\mathrm{Quantile}}_{25}\left( {{\mathrm{S}}_{\mathrm{S}}} \right)} \right).$$

Where SS represents the correlation of known S phase genes with S phase score and SG2M represents the correlation of known S phase genes with G2M score. In the same manner, the G2M cut-off is:

$${\mathrm{Max}}\left( {{\mathrm{Med}}\left( {{\mathrm{G2M}}_{{\mathrm{G2M}}}} \right) - {\mathrm{Med}}\left( {{\mathrm{G2M}}_{\mathrm{S}}} \right),{\mathrm{Quantile}}_{25}\left( {{\mathrm{G2M}}_{{\mathrm{G2M}}}} \right)} \right).$$

Genes with a correlation to either S or G2M scores above the respective threshold were considered as cell cycle genes and therefore excluded from the variable genes removed. Additional filtering was carried out when processing the data set in Fig. 3a. We removed ribosomal protein genes and unnamed transcripts by filtering genes symbols starting with “RP” from variable genes. The first eight principal components were used to calculate dimensionality reduction, and a resolution of 1.35 was used to define clusters.

When processing early/late data sets shown in Supplementary Fig. 5b, c, we removed heat shock protein genes, as defined by GO:0006986 (response to unfolded protein) as well as ribosomal protein genes, based on the term GO:0022626 (cytosolic ribosome). Lastly, we removed genes residing on chromosome Y, as well as female-exclusive XIST and TSIX. For the early/late comparison of organoid 51 and 80, the first six and ten principal components, respectively, were used to calculate dimensionality reduction, and a resolution of 0.6 and 0.5, respectively, was used to define clusters.

Differential expression analysis was performed comparing each population to all other populations originating from the same organoid using the Wilcoxon test with 1.8-fold expression cut-off, and 5% Bonferroni multiple testing corrected statistical significance cut-off. For enrichment analysis, the R package clusterProfiler version 3.12 was used^84^.

Cell-type identification was performed using SingleR version 1.0.1^85^. The expression profile of each single cell was correlated to Human Primary Cell Atlas-derived microarray expression data, containing 713 samples representing 38 main cell types. Four major cell types were present in the data set, neuroepithelial cells, epithelial cells, and stromal cells consisting of MSC, fibroblasts, chondrocytes and smooth muscle cells. Cells of each cluster were assigned to cell types by majority vote.

All the scripts used are available at https://bitbucket.org/princessmaximacenter/kidney_organoid_biobank/src/master/

### Bulk RNA sequencing

The total RNA was extracted from organoids and tissue using Trizol reagent (Invitrogen), and quality was checked with Bioanalyzer2100 RNA Nano 6000 chips (Agilent, Cat. 5067-1511). Sequencing libraries were prepared using the NEBNext® Ultra™ RNA Library Prep Kit (New England Biolabs) according to the manufacturer’s protocol. Paired-end sequencing was performed on the Illumina HiSeq (PE150) by Novogene (Hong Kong). 3′-adaptors were trimmed with cutadapt version 1.16 and resulting sequences shorter than 20 bp were discarded. The remaining reads were mapped to hg38 Patch 10 using STAR version 2.6.1. Read assignment was performed with featureCounts version 1.5.2 as described for scRNA-seq. The resulting raw count table was converted to TPM before downstream analysis.

The analysis of the bulk RNA-seq was done as described for the single-cell analysis, with the following modifications: no cell filtering was imposed; transcript counts were normalised to 1 million; regression of the growth environment was applied; the first four PCs were used for the graph-based clustering; a twofold change was used for differential expression using the bimodal test with the same significance cut-off as above. The STAR-fusion (version 1.4.0) pipeline was used to identify chimeric reads and call fusion transcripts using default parameters. Non-ref splice hits were filtered out, and a FFPM cut-off was set at 1. Plots were generated using the Chimeraviz R package (version 3.8).

### Western blot

Western blot on organoids was performed as described^63^. P53 clone DO-1 (sc-126, Santa Cruz Biotechnology, 1:1000) and GAPDH (ab-9485, Abcam, 1:1000) were used as primary antibodies.

### Drug screening

Organoids were harvested and washed in ice-cold AdDF+++. Next, organoids were filtered using a 70-µm nylon cell strainer (Falcon) and resuspended in 5% BME in kidney organoid medium. Subsequently, ~500 organoids were plated using the Multi-drop^TM^ Combi Reagent Dispenser on repellent black 384-well plates (Corning) to which medium with compounds were added (six different concentrations) using either the Caliper Sciclone—Robotic Liquid Handling robot or the Tecan D300e Digital Dispenser. Drugs and positive (staurosporin (Sigma-Aldrich)) and negative (DMSO) controls were dispensed such that final DMSO concentration was 1% in all wells. Four technical replicates were included in each experiment. Five days after adding the drugs, ATP levels were measured using CellTiter-Glo® 2.0 (Promega) according to the manufacturer’s instructions on a Spark microplate reader (Tecan). The results were normalised to DMSO vehicle (100%). For the validation assays, nine concentrations and four technical replicates were included per compound per experiment. Data were analysed with software GraphPad Prism v7.04.

### Reporting summary

Further information on research design is available in the Nature Research Reporting Summary linked to this article.

## Supplementary information

Supplementary Information

Description of Additional Supplementary Files

Supplementary Data 1

Supplementary Data 2

Supplementary Movie 1

Supplementary Movie 2

Reporting Summary

## Data Availability

The sequencing data have been deposited to the European Genome-Phenome Archive (www.ebi.ac.uk/ega/) under accession numbers EGAD00001005319 and EGAD00001005318. DNA methylation data have been deposited to GEO (www.ncbi.nlm.nih.gov/geo/) under accession number GSE137544. COSMIC SigProfiler database [https://www.synapse.org/#!Synapse:syn11967914] has been used for mutational signatures analysis. Filtering scripts used mutational signatures analysis are available at https://github.com/UMCUGenetics/SNVFI and https://github.com/ToolsVanBox/INDELFI.

## Source Data

Source Data
