# Supplementary material for: An organoid biobank for childhood kidney cancers that captures disease and tissue heterogeneity
Source: Nat Commun. 2020 Mar 11;11:1310. doi: 10.1038/s41467-020-15155-6 (PMC7066173; doi:10.1038/s41467-020-15155-6)
Supplement: Supplementary file 1 — Supplementary Information [file 41467_2020_15155_MOESM1_ESM.pdf]

Supplementary information

An Organoid Biobank for Childhood Kidney Cancers that Captures Disease and Tissue Heterogeneity

Calandrini, Schutgens et al.

Supplementary Fig. 1

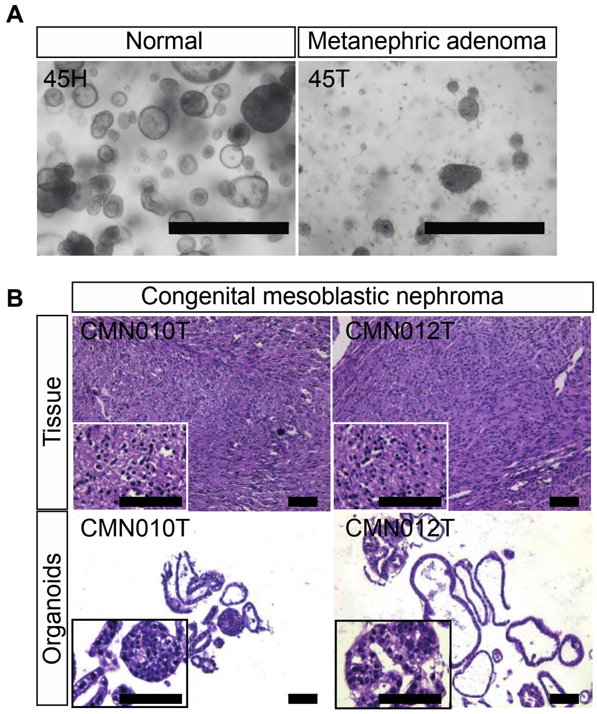

Supplementary Figure 1. Phenotypic characterization of paediatric kidney cancer organoids.

(A) Representative brightfield microscopy images of normal kidney tissue-derived organoids and matching metanephric adenoma organoids (n=3). Scale bar: 1mm. **(B)** H&E staining on tissue (top) and matching organoids (bottom) derived of two congenital mesoblastic nephromas (n=3). Scale bars: 100 µm.

Supplementary Fig. 2

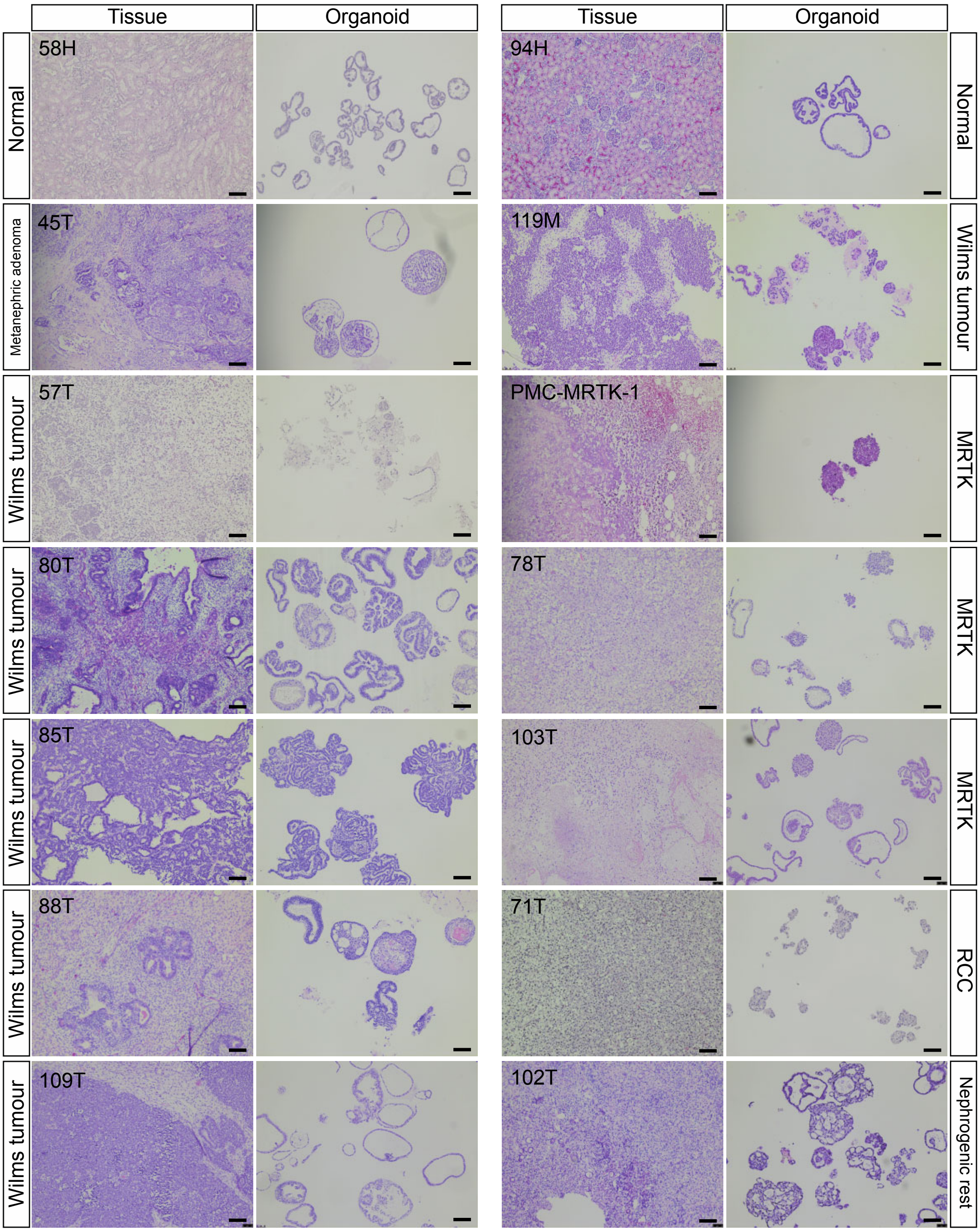

Supplementary Figure 2. Histology of paediatric kidney cancer organoid lines resembles primary tumour tissue.

H&E staining on tissue (left panels) and matching organoids (right panels) derived of the indicated tumour types (n=3). Additional cases can be found in Fig. 2a and Supplementary Fig. 1b. H, healthy; T, primary tumour; M, metastasis. Scale bars: 100 µm.

Supplementary Fig. 3

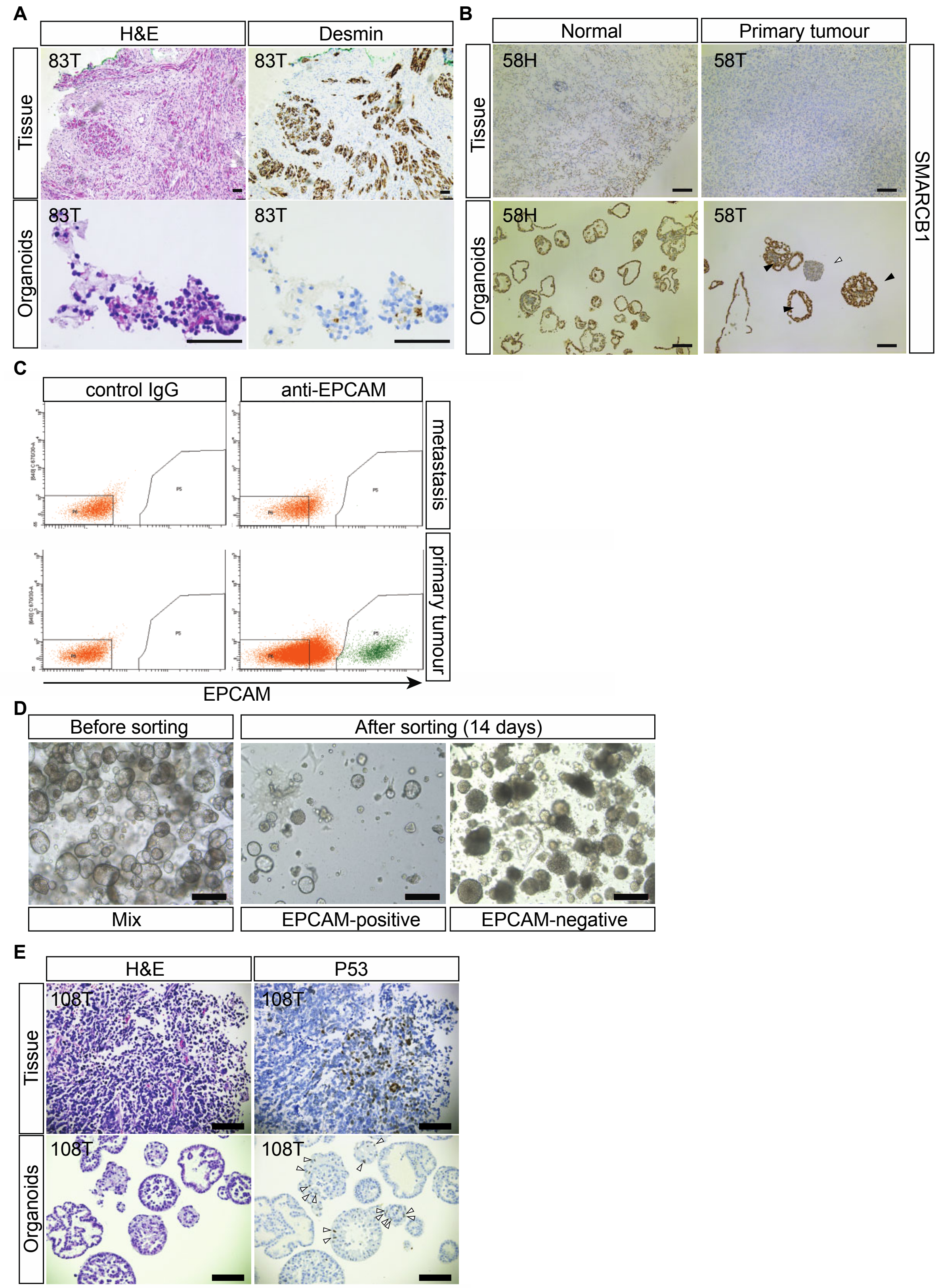

Supplementary Figure 3. Confirmation of tumour origin of organoids.

(A) Representative H&E (left) and desmin (right) stainings on a Wilms tumour (tissue (top) and organoids (bottom)) with rhabdomyomatous differentiation (n=3). Scale bars: 50 µm. **(B)** SMARCB1 stainings on normal kidney (left) and MRTK tumour (right) tissue (top) and organoids derived thereof (bottom), demonstrating the presence of SMARCB1-positive normal kidney tissue-derived organoids in the tumour organoid culture (n=3). Of note, immune cells stain positive for SMARCB1 in MRTK tissue. Open arrowhead indicates SMARCB1-negative MRTK organoids; closed arrowheads indicate SMARCB1-positive normal kidney organoids. Scale bars: 100 µm. **(C)** EPCAM-negative MRTK cells are purified from a mixed culture (Supplementary Fig. 3b) using FACS. Gating strategy are exemplified in Supplementary Fig. 13a. **(D)** Representative brightfield microscopy images of MRTK organoid cultures before (left) and after FACS-purification of EPCAM-positive (middle) and -negative (right) cells (n=2). Scale bar: 200 µm. **(E)** H&E (left) and P53 (right) on an RCC expressing mutant P53 (n=3). Arrowheads indicate several P53-positive nuclei. Scale bars: 100 µm.

Supplementary Fig. 4

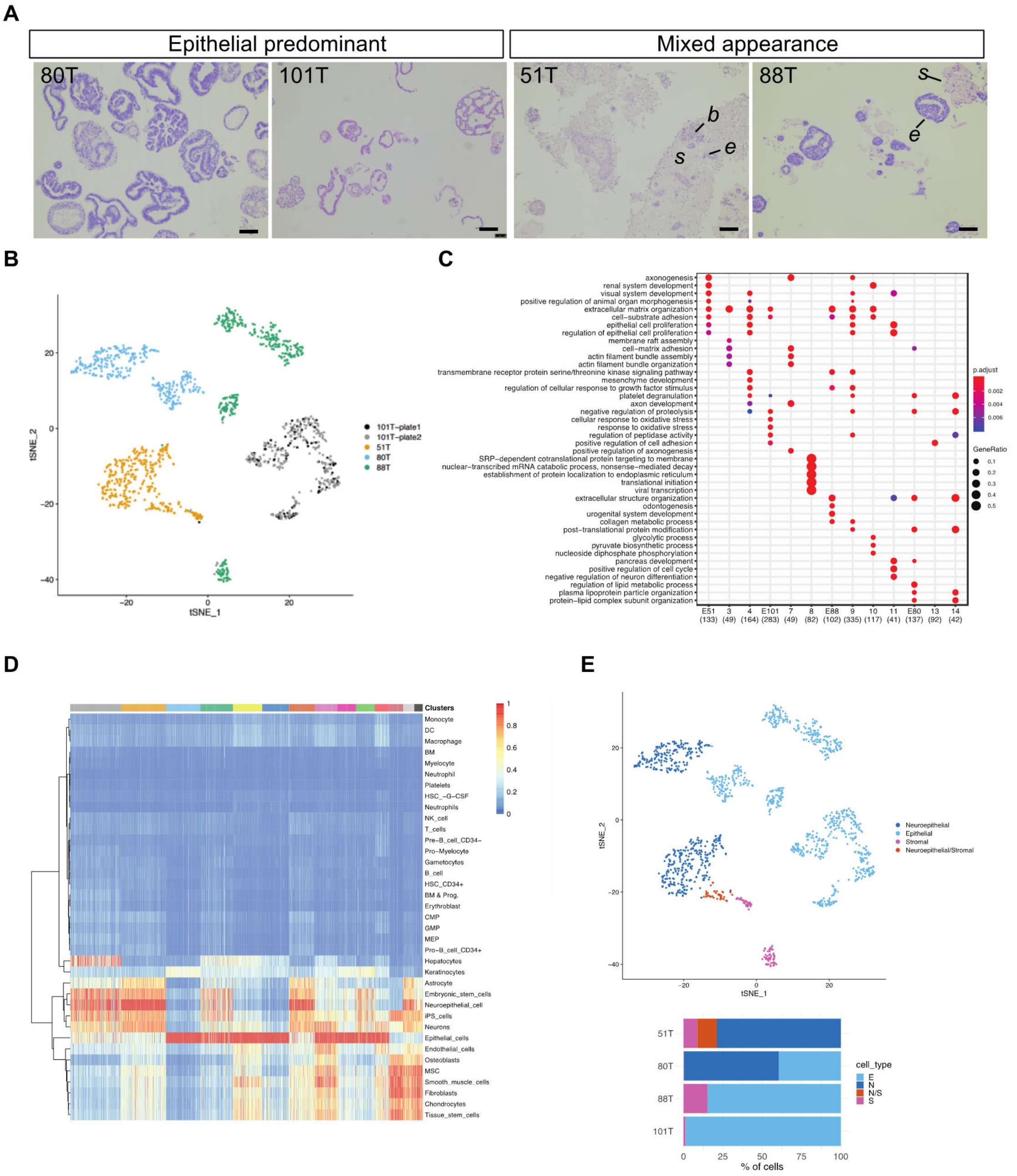

Supplementary Figure 4. Cellular heterogeneity within Wilms tumour organoid cultures. **(A)** H&E staining on the four Wilms tumour organoid cultures that were used for the scRNA-seq experiment. Two predominantly epithelial cultures (80T, 101T) and two with apparent different cell types (51T, 88T) b, blastemal; e, epithelial; s, stromal (n=3). Scale bars: 100 µm. **(B)** t-SNE representation of single cells from four Wilms tumour organoid lines (51T, 80T, 88T, 101T). Cells are coloured by plate of origin. For one organoid (101T), cells were sorted in two plates, which were processed separately. Cells from these two plates cluster together, thereby excluding technical batch effects. **(C)** Gene Ontology- Biological Process (GO BP) enrichment analysis of higher expressed genes between organoid source or cell population, as shown in Fig. 3a. The Benjamini-Hochberg adjusted p-value levels are color-coded, while the size of the dots represents the ratio of differentially expressed genes belonging to the specific GO BP term. **(D)** Unbiased cell type identification with SingleR classification using the Human Primary Cell Atlas Reference. Heatmap showing the Spearman correlation scores of the expression profile of every single cell with that of each reference sample. The four major cell types present in the dataset are: Neuroepithelial cells, epithelial cells, and a stromal component consisting of MSC, fibroblasts, chondrocytes, and smooth muscle cells. **(E)** t-SNE map showing the four major cell types and a bar plot depicting the fraction cell types for each organoid.

Supplementary Fig. 5

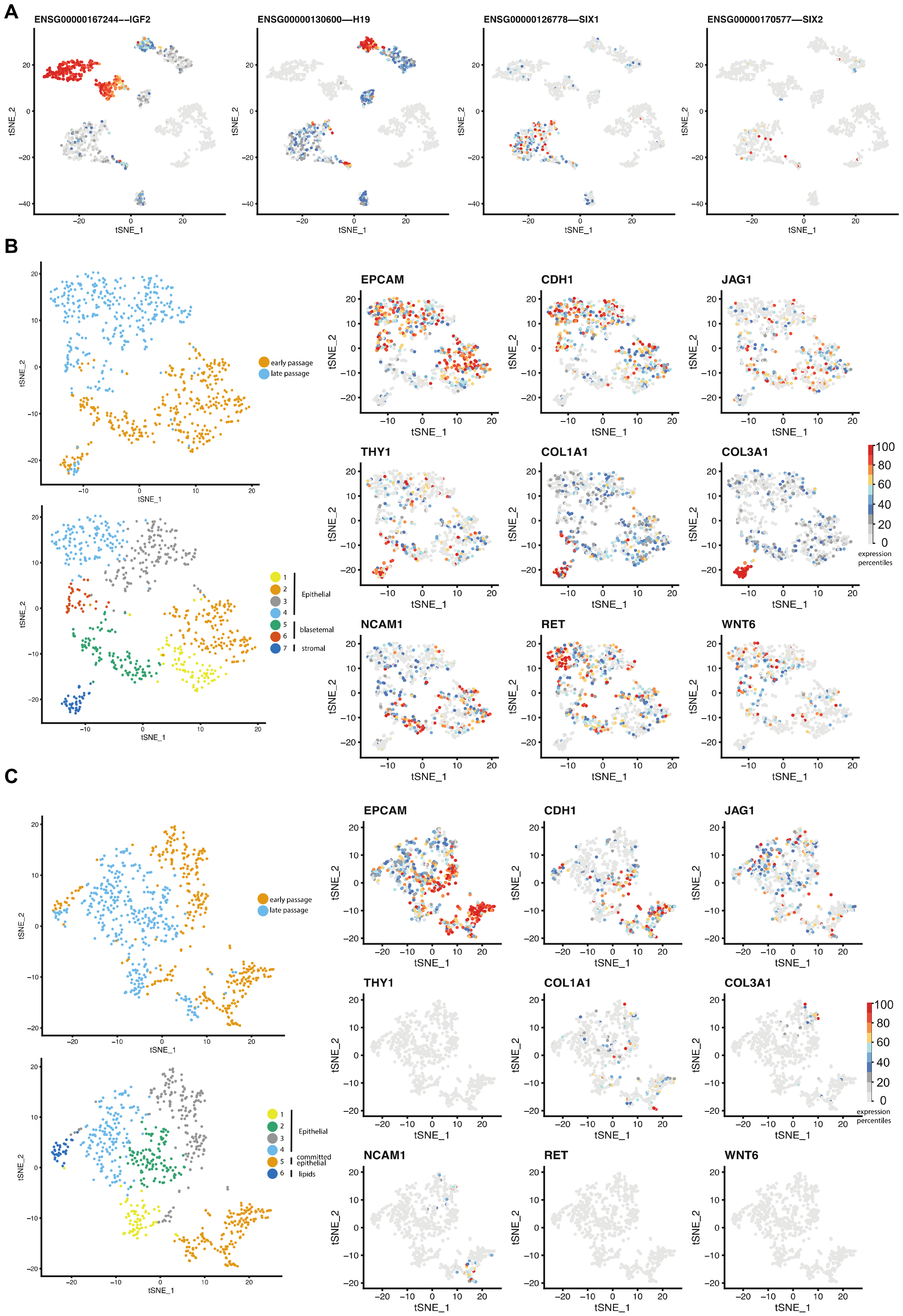

Supplementary Figure 5. Different cell types in Wilms tumour organoids can be retained over time.

(A) Additional t-SNE maps showing the color-coded logged expression levels of several markers demonstrating that different cell types are present in 51T and 88T, whereas 80T and 101T organoids primarily consist of different epithelial sub-populations, which is in line with their histological appearance (additionally see Fig. 3b and Supplementary Fig. 4). **(B)** and **(C)** t-SNE representation of single cells from two different passages (early and late (5 – 6 passages in between, representing approximately 3 months of culturing)) of two Wilms tumour organoid lines (51T (B), 80T (C)). Cells are color-coded by early/late passage (top left) or clustering results (bottom left). t-SNE maps showing the color-coded logged expression levels of several markers (right panels) for each population demonstrating that different cell types are retained over time. For instance, population number 7 in 51T consists of early as well as late passage cells. These results are in line with the FACS experiments for different cell types (Supplementary Fig. 6a).

Supplementary Fig. 6

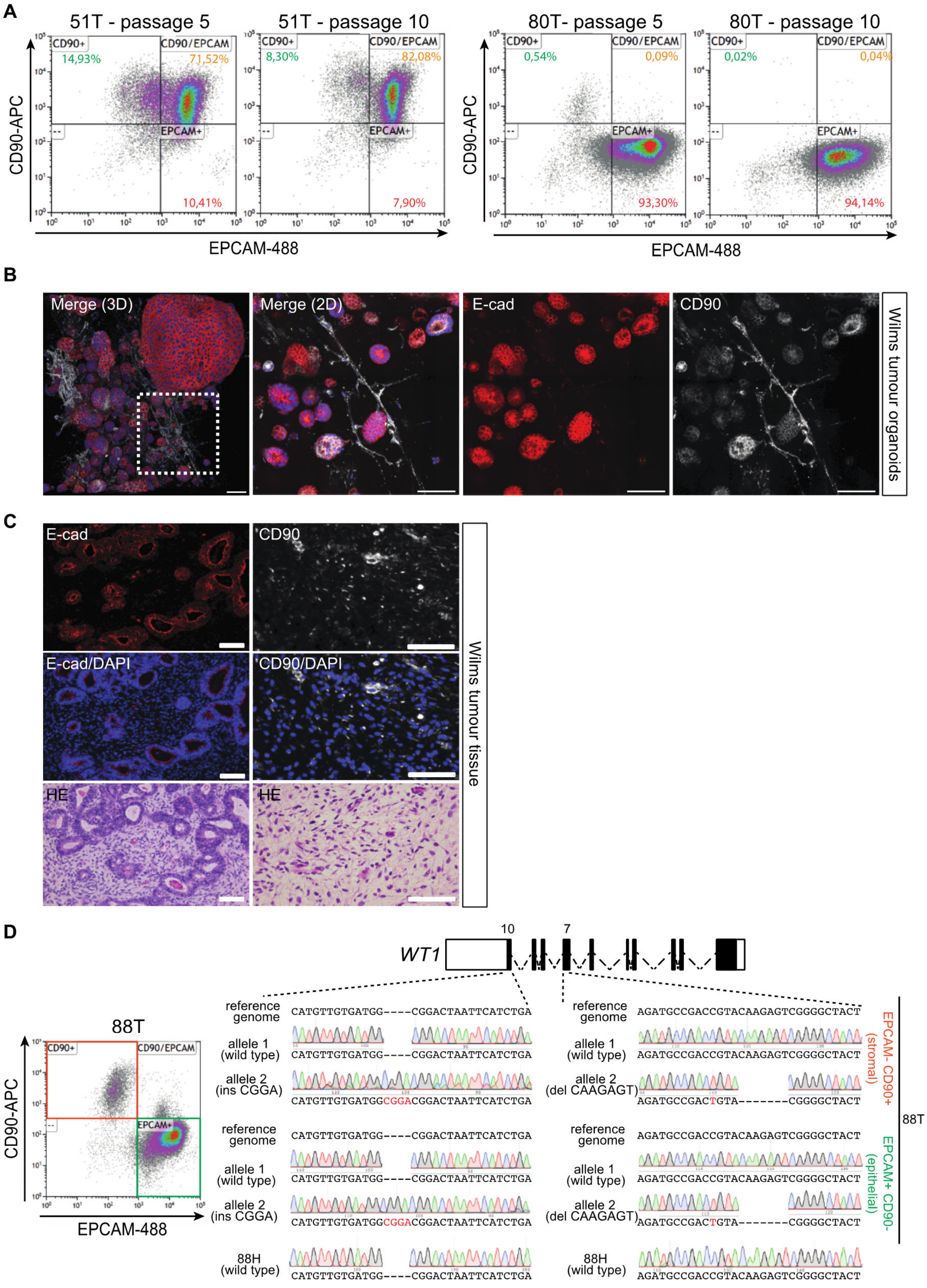

Supplementary Figure 6. Cellular heterogeneity within Wilms tumour organoid cultures visualized with high resolution 3D imaging.

(A) FACS analyses of two different passages (early and late (5 passages in between, representing approximately 3 months of culturing)) of two Wilms tumour organoid lines (51T (tri-phasic), 80T (epithelial predominant). EPCAM (epithelial marker) and CD90 (THY1, stromal marker) were used to quantify epithelial (EPCAM single positive), stromal (CD90 single positive, and "blastemal"-like cells (expressing both markers (i.e. EPCAM/CD90 double positive)). Gating strategy are exemplified in Supplementary Fig. 13b. **(B)** High resolution 3D imaging of 88T Wilms tumour organoids (containing stromal cells based on scRNA-seq (Fig. 3a, b) immunolabeled for E-cadherin (E-cad; red) and CD90 (white) (n=2). Scale bars, 100 µm (left panel) and 50 µm (other panels). **(C)** Immunofluorescence imaging on 88T Wilms tumour tissue sections immunolabeled for E-cadherin (E-cad; red), CD90 (white), and DAPI (blue) (n=2). Scale bars 100 µm. **(D)** Based on marker expression, epithelial (EPCAM-positive) and stromal (CD90-positive) cells were purified using FACS from 88T (left panel). Purified populations were subsequently genotyped for the presence of *WT1* mutations (as revealed by WGS on bulk organoid culture and matching tumour tissue (Fig. 4a)). *WT1* (exon 7, exon 10) mutations are present in epithelial as well as stromal cells, thereby demonstrating that both are tumour cells. *WT1* mutations are absent in matching normal kidney-derived organoids (88H, bottom).

Supplementary Fig. 7

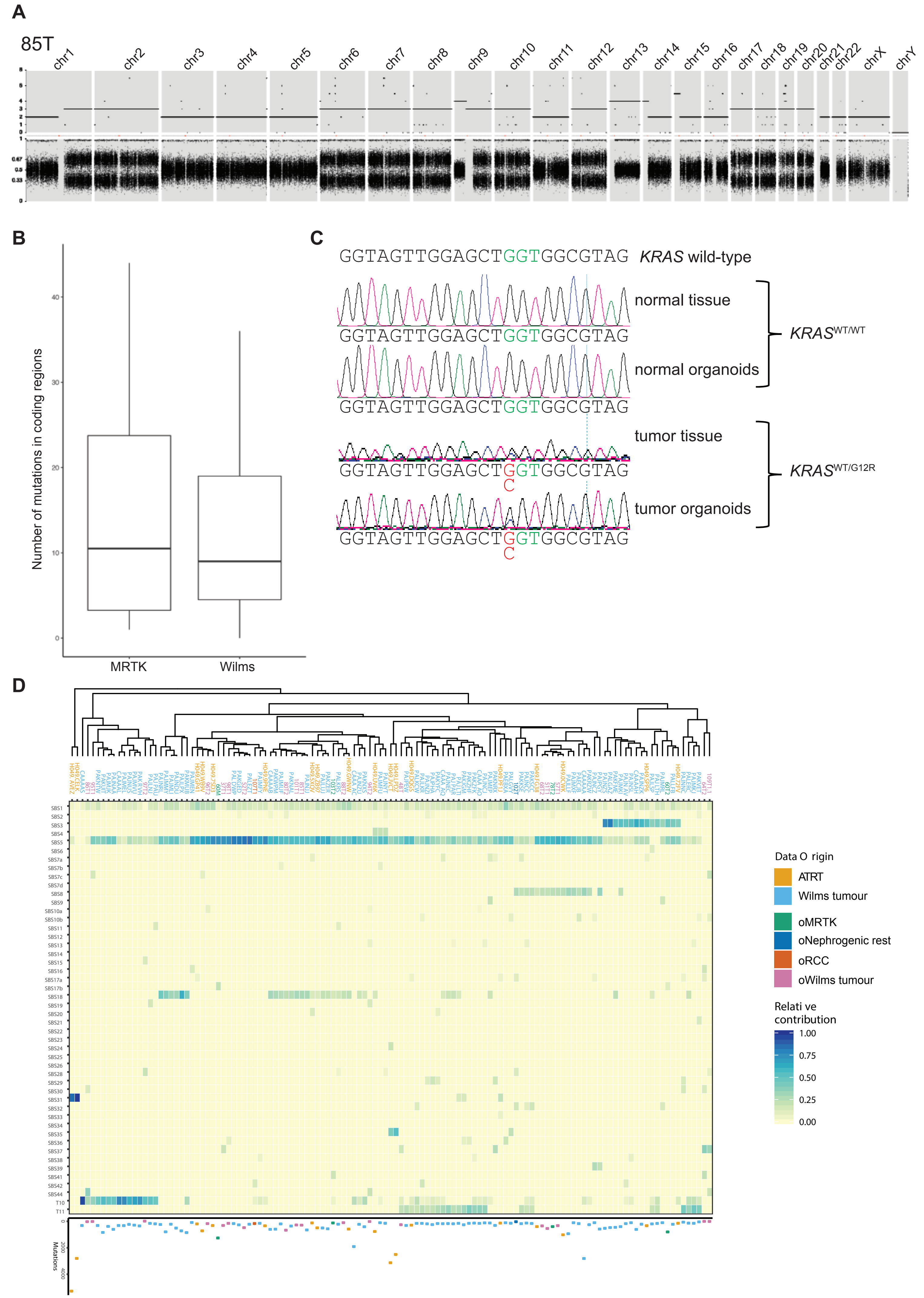

Supplementary Figure 7. Genetic characterization of paediatric kidney cancer organoids.

(A) Example of copy number alteration (top) and B Allele Frequency (BAF) plots (bottom) for one of the Wilms tumour organoid lines (85T). **(B)** Boxplot depicting the average number of mutations in coding regions in all primary tumour-derived MRTK (left) and Wilms tumour (right) organoids. MRTK: minima=1, maxima=44, mean=16.5, 1st Qu=3.25, 3rd Qu=23.75, highest whisker =44, lower whisker =1. Wilms: minima=0, maxima=36, mean=12.28, 1st Qu=4.5, 3rd Qu=19, highest whisker =36, lower whisker =0. **(C)** Targeted amplification and subsequent Sanger sequencing of part of the *KRAS* gene reveals a heterozygous *KRAS*^{G12R} mutation in metanephric adenoma tissue and organoids derived thereof. This mutation is absent in matching normal tissue and organoids. **(D)** Hierarchical clustering of samples based on the relative contributions of signatures (top, dendrogram), the relative contributions of the COSMIC signatures (middle, heatmap) and the mutation load in each sample (bottom, dot plot). The colours of the sample names and dot plot indicate the cancer types (ATRT: atypical teratoid rhabdoid tumour, MRTK: malignant rhabdoid tumour of the kidney, RCC: renal cell carcinoma) with the preceding "o" indicating they are from organoids.

Supplementary Fig. 8

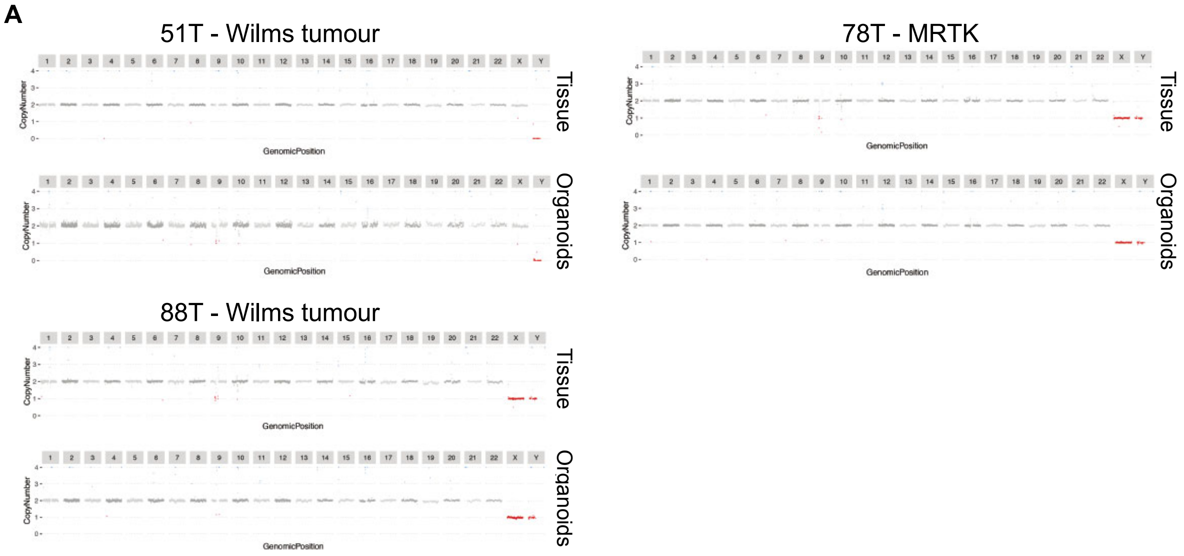

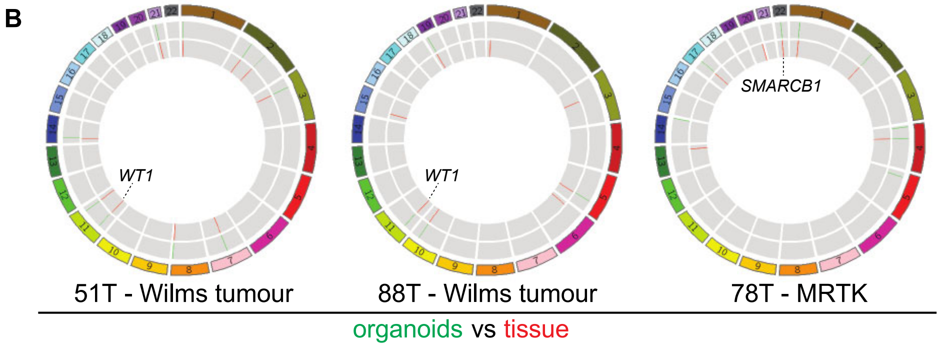

Supplementary Figure 8. Paediatric kidney cancer organoids reflect the genetic landscape of parental tumour tissues.

(A) Genome-wide CNAs (karyograms) and **(B)** coding gene mutations (circos plots) in matching tumour tissue vs organoid pairs reveal that organoids recapitulate the genetic landscape of the tissue they were derived from.

Supplementary Fig. 9

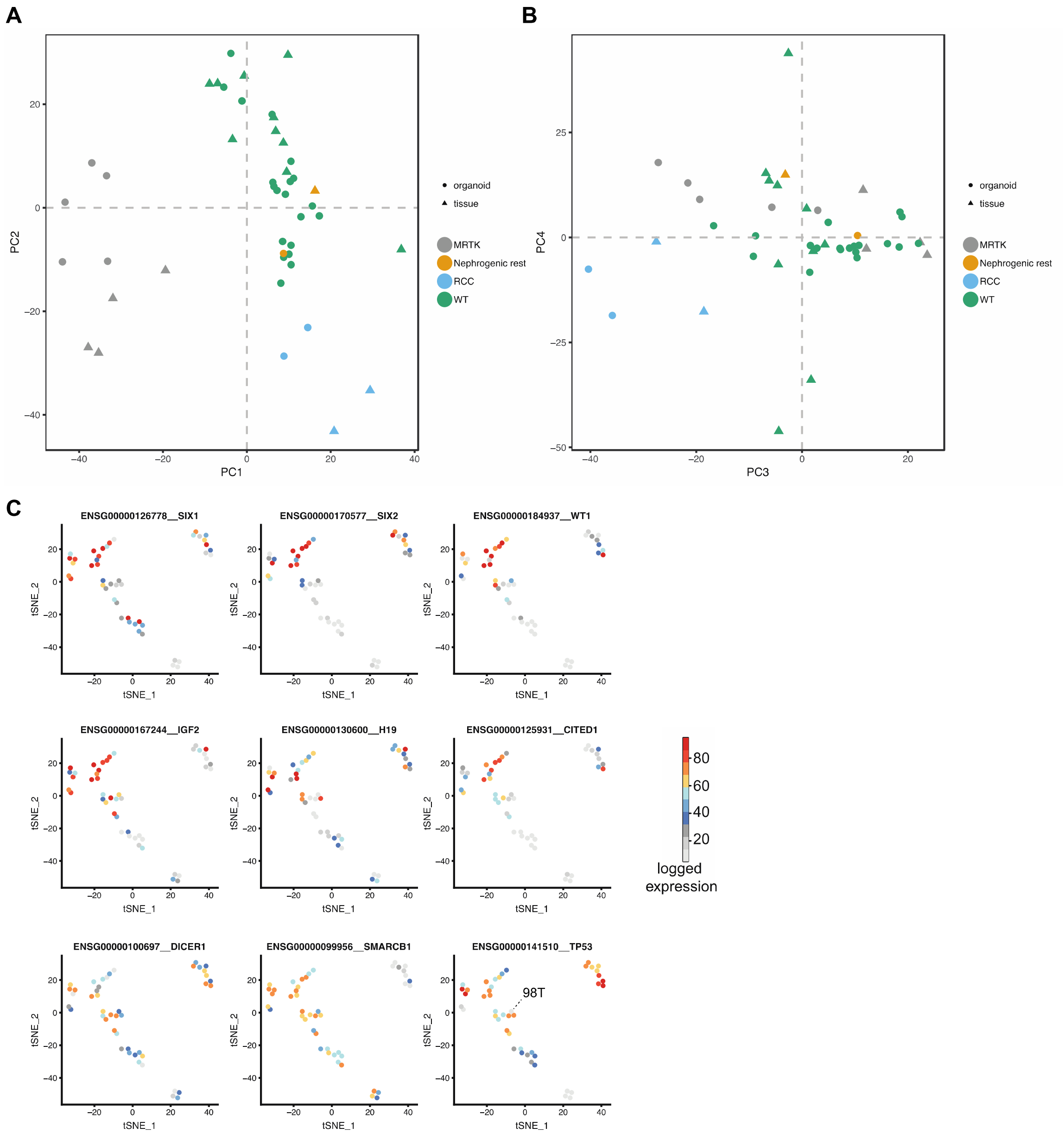

Supplementary Figure 9. Transcriptome profiling of paediatric kidney cancer organoids using RNA-seq.

(A), **(B)** Principal component analysis of the tumour organoid and tissue samples without regressing batch effects separate growth conditions and tumour type. **(C)** t-SNE maps, as in Fig. 5a, showing expression levels of additional marker genes to the ones shown in Fig. 5b.

Supplementary Fig. 10

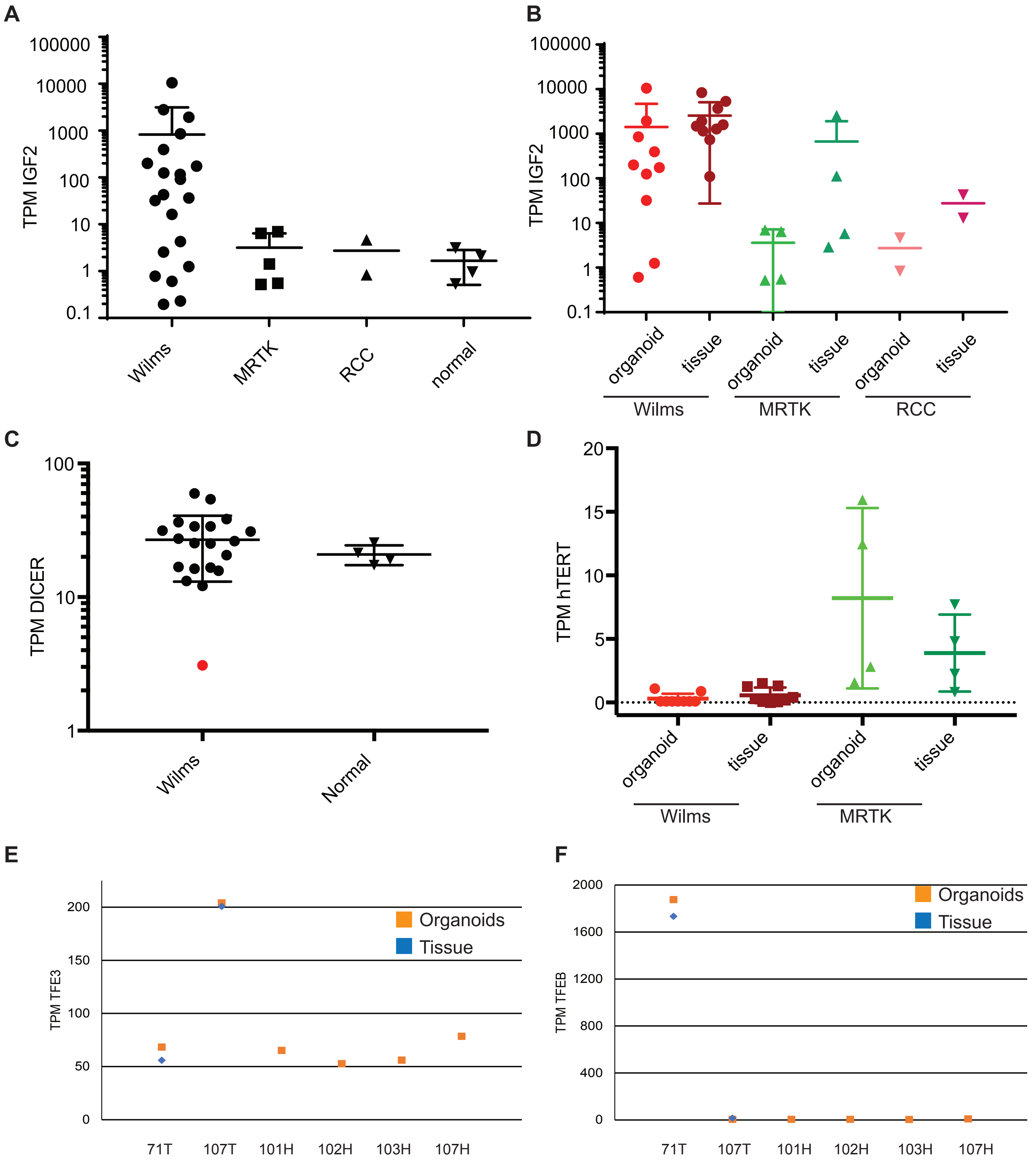

Supplementary Figure 10. RNA-seq reveals common gene expression profiles of paediatric kidney tumours.

(A) Normalized *IGF2* transcripts per million (TPM) values in the indicated paediatric kidney tumour organoids. Data are represented as mean values ± SD; for RCC group, data is represented as mean values (Wilms: n=21, MRTK: n=5, RCC: n=2, normal: n=4). **(B)** Normalized *IGF2* TPM values in the indicated paediatric kidney tumour organoids and parental tumour tissues. Data are represented as mean values ± SD; for RCC organoid and tissue data are represented as mean values (Wilms organoid: n=10, MRTK organoid: n=4, RCC organoid: n=2, Wilms tissue: n=10, MRTK tissue: n=4, RCC tissue: n=2). **(C)** Normalized *DICER1* TPM values in Wilms tumour and normal tissue-derived organoids. Red dot represents cystic Wilms tumour-derived organoid line (WT003T). Data are represented as mean values ± SD (Wilms: n=20, normal: n=4) **(D)** As in (B), but for *hTERT*. Data are represented as mean values ± SD (Wilms organoid: n=9, MRTK organoid: n=4, Wilms tissue: n=9, MRTK tissue: n=4). **(E)** Normalized *TFE3* TPM values in the indicated paediatric tRCC-derived (71T, 107T) and normal tissue-derived organoids **(F)** Normalized *TFEB* TPM values in the indicated paediatric tRCC-derived (71T, 107T) and normal tissue-derived organoids. Source data are provided as a Source Data file.

Supplementary Fig. 11

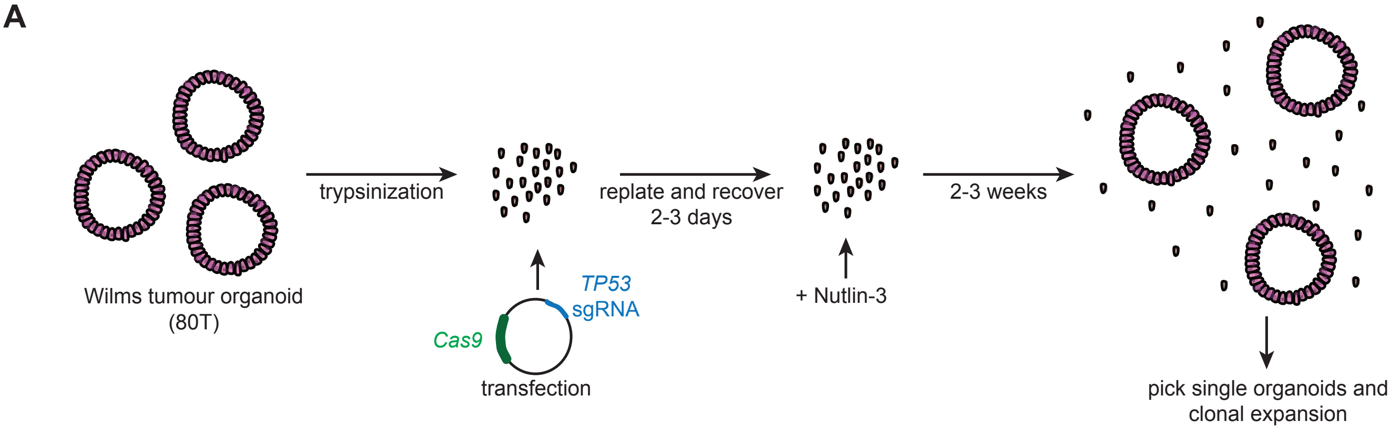

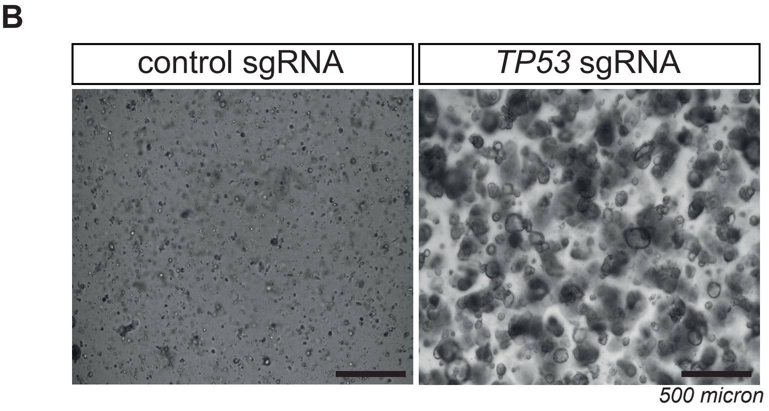

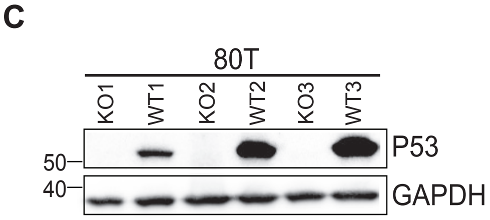

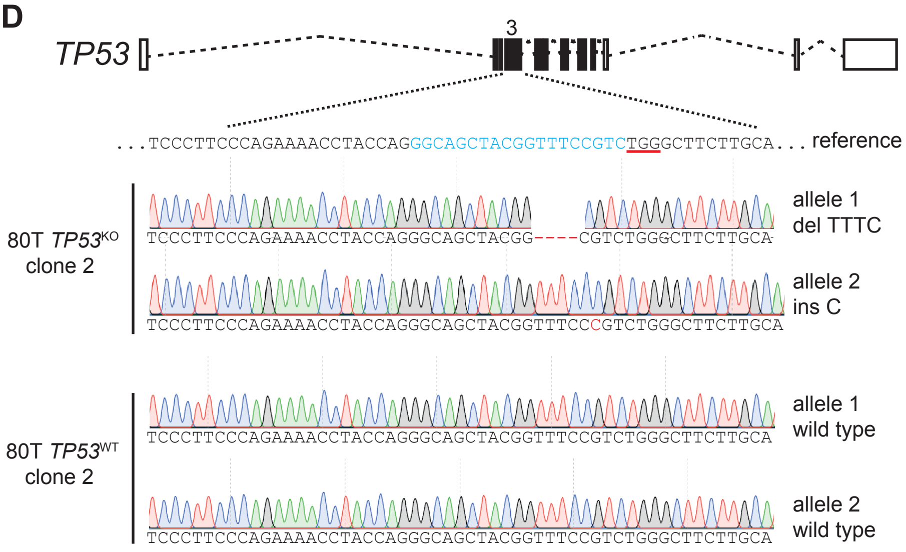

Clone	*TP53* mutation
TP53^{WT} clone 1	-
TP53^{WT} clone 2	-
TP53^{WT} clone 3	-
TP53^{mut} clone 1	Allele1 17:7579360 del1bp<br>Allele2 17:7579359_7579360 insC
TP53^{mut} clone 2	Allele1 17:7579360_7579363 del4bp<br>Allele2 17:7579359_7579360 insC
TP53^{mut} clone 3	Allele1 17:7579360 del1bp<br>Allele2 17:7579359_7579360 insC

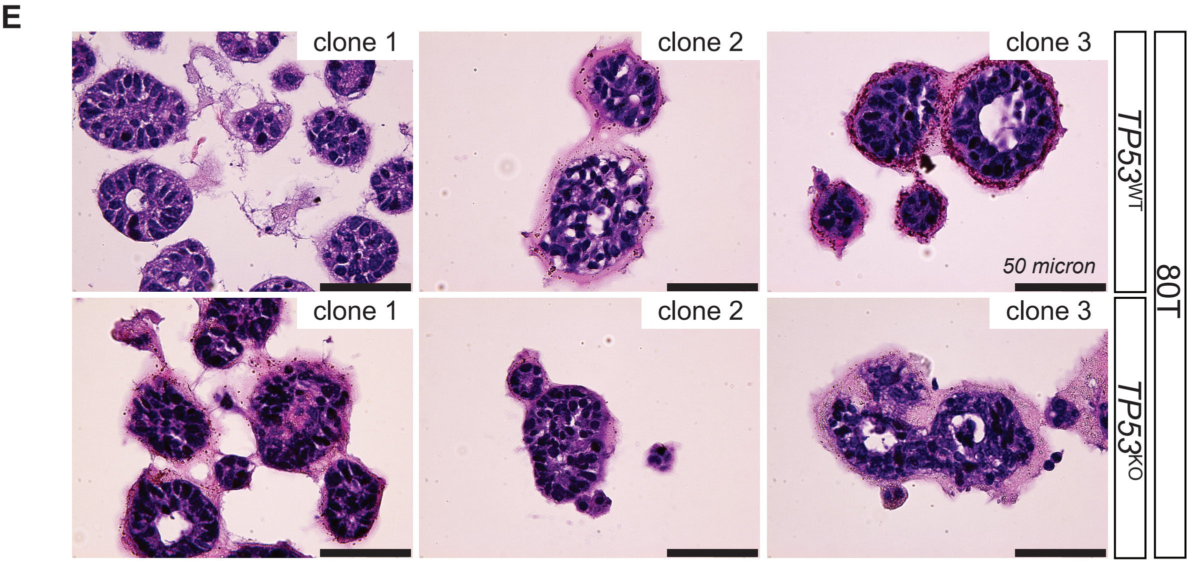

Supplementary Figure 11. Gene editing of Wilms tumour organoids.

(A) Scheme depicting the process of generating *TP53* mutant Wilms tumour organoids. **(B)** Representative brightfield images of 80T Wilms tumour organoids transfected with either control sgRNA (left) or TP53 sgRNA (right) and grown in medium containing nutlin-3 to select for *TP53* mutant cells. Scale bars 500 µm. **(C)** Western blot analysis of P53 expression in clonally expanded 80T (*TP53* wildtype (WT)) and 80T-*TP53*^{KO} organoids. The experiment has been performed for three independent clones of each genotype (WT 1-3; KO 1-3). **(D)** Sequence analysis of the targeted *TP53* exon. PCR amplification products of the mutated alleles were obtained using primers flanking the targeted exon. Subsequent sequencing revealed indels at the expected locations. PAM sequence is underlined in red in wild-type sequence. **(E)** H&E staining on three independent 80T *TP53* wild-type and three independent 80T-*TP53*^{KO} clones. No apparent differences can be observed. Images are representative of n=2 independent experiments. Scale bars 50 µm. Source data are provided as a Source Data file.

Supplementary Fig. 12

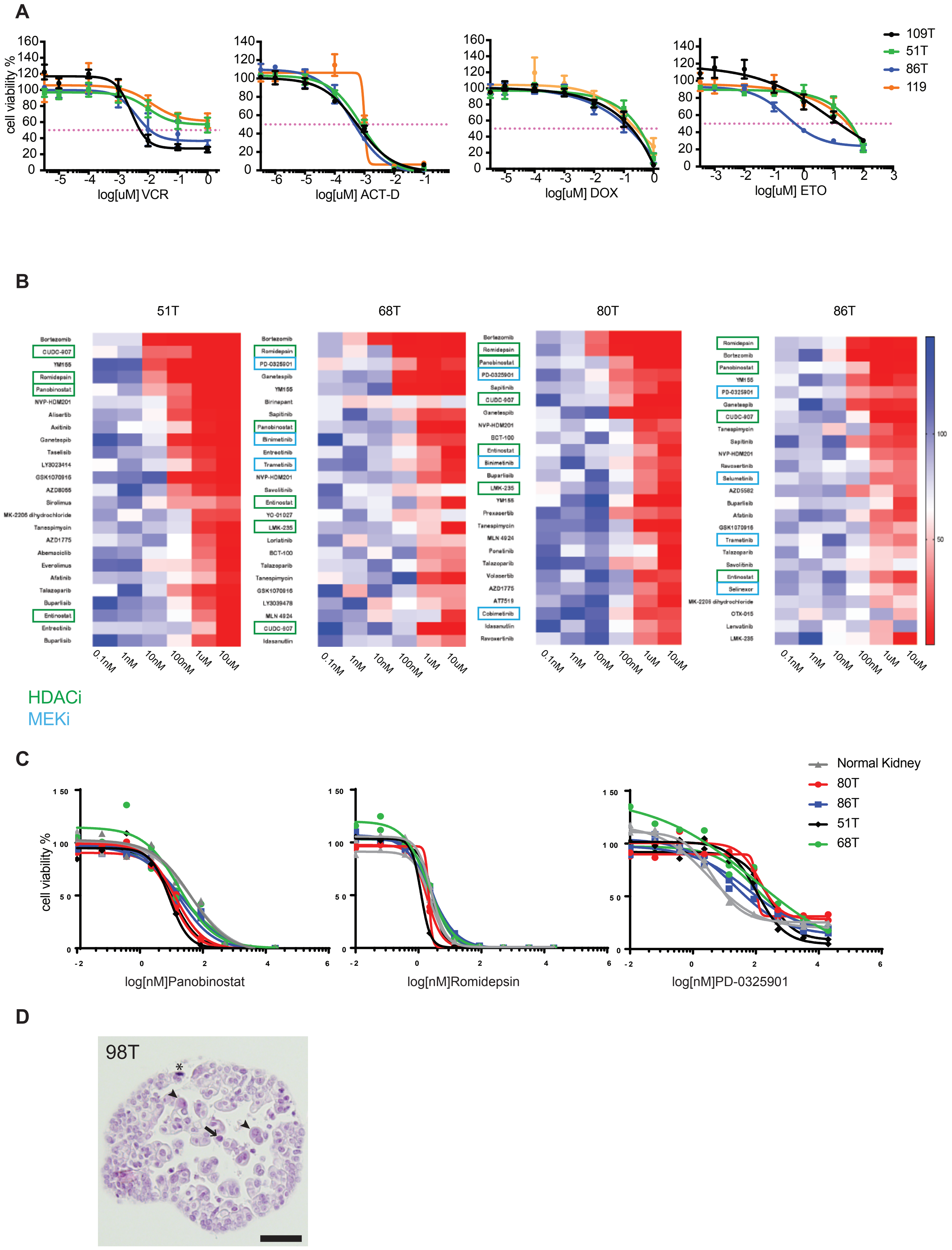

Supplementary Figure 12. Organoid drug screens reveal patient-specific drug sensitivities.

(A) Dose-response curves of vincristine (VCR), actinomycin D (ACT-D), doxorubicin (DOX,), and etoposide (ETO) of the indicated Wilms tumour organoid lines. Error bars represent SEM of three independent experiments (each individual experiment includes technical quadruplicates). **(B)** Compound screens (approximately 150 compounds) were performed on the indicated Wilms tumour organoid cultures. Depicted are the top 25 calculated area under the estimated dose-response curve (AUC) per organoid culture. Multiple MEK (blue) and HDAC (green) inhibitors are shared between the four cultures. Colour scale blue to red indicates decreasing ATP levels relative to DMSO control. **(C)** Dose-response curves of Panobinostat, Romidepsin, and PD-0325901 of the indicated Wilms tumour organoid lines. Curves with the same colour represent independent experiments. Each individual point represents the average of quadruplicate measurements. **(D)** H&E staining on an anaplastic Wilms tumour-derived organoid culture (98T) showing two anaplastic features: hyperchromatic nuclei (arrow) and enlarged nuclei (arrowhead). In addition, a mitotic figure is present (*). No atypical mitoses were detected (n=3). Scale bar: 50 µm. Source data are provided as a Source Data file.

Supplementary Fig. 13

A

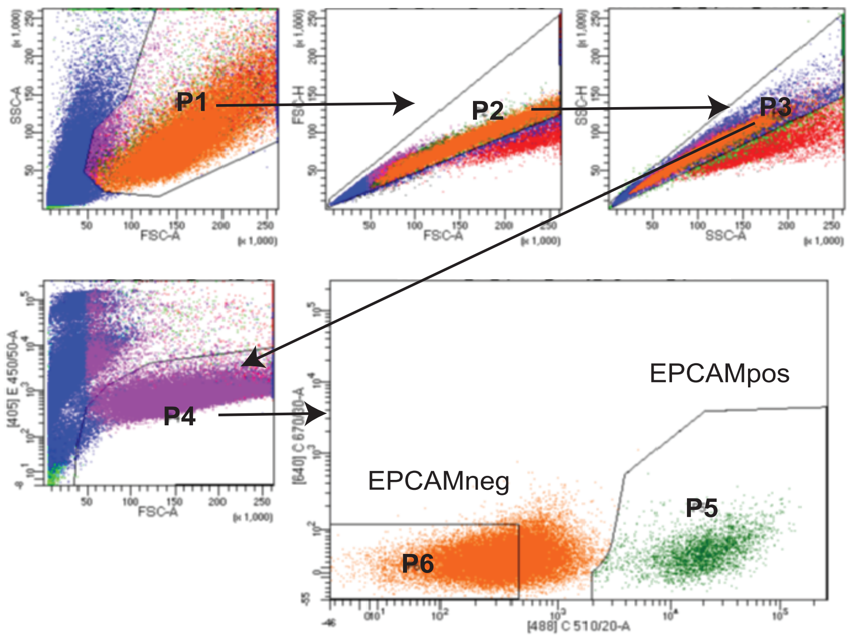

B

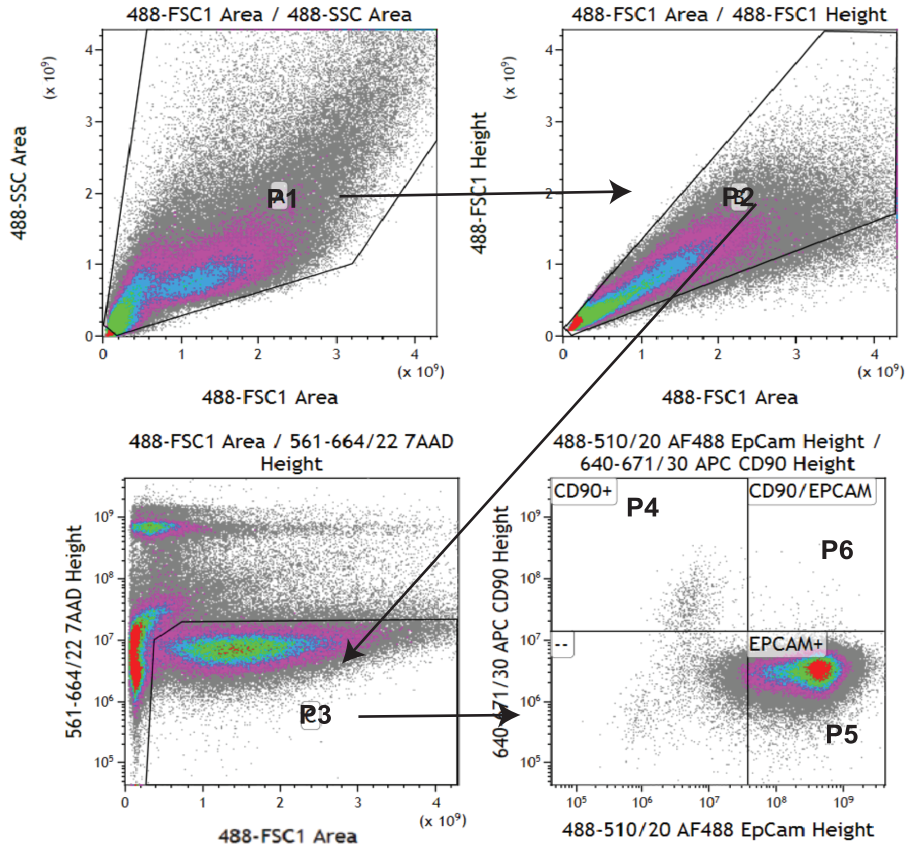

Supplementary Figure 13. Gating strategies.

(A) Example of gating strategy applied to purify EPCAM negative and positive organoid populations (Supplementary Fig. 3c). **(B)** Example of gating strategy applied to quantify single (EPCAM or CD90) or double positive (EPCAM/CD90) organoid populations over time (Supplementary Fig. 6a). For CD90 and EPCAM gating, we used unlabelled cells of interest, single-color controls (UltraComp beads ThermoFisher, or partially killed cells of interest) followed by automatic compensation matrix calculations (fluorescence-minus-one (FMO) controls for each marker).

Line	Gender	Year of birth	Diagnosis	Source	Low/intermediate/high risk	Pre-op treatment	Histopathology
37	F	2013	WT	primary	intermediate	6 weeks VAD	Regressive
45	M	2014	Metanephric adenoma	primary	n/a	4 weeks AV	n/a
48	F	2014	WT	primary	intermediate	6 weeks AV	Bilateral, material from 1 kidney.
51	F	2013	WT	primary	intermediate	4 weeks AV	Mixed type (blastema/epithelium/stroma)
52	F	2010	WT	primary	intermediate	4 weeks AV	Mixed type (blastema/stroma/epithelium)
57	F	2009	WT (Beckwith Wiedemann)	primary	intermediate	4 weeks AV	Mixed type (blastema/epithelium)
58	M	2016	MRTK	primary	n/a	4 weeks AV	n/a
60	F	2015	MRTK	primary	n/a	5 weeks AV	n/a
60	F	2015	MRTK	Met (lymph node)	n/a	5 weeks AV	n/a
64	F	2015	WT	primary	intermediate	4 weeks AV	Mixed type
68	M	2011	WT	primary	intermediate	4 weeks AV	Mixed type (blastema/stroma/epithelium)
71	F	2011	RCC	primary	n/a	4 weeks AV	n/a
78	M	2016	MRTK	primary	n/a	2 weeks AV	n/a
80	F	2013	WT	primary	intermediate	4 weeks AV	Mixed type (epithelium/stroma/minimal blastema)
83	F	2015	WT	primary	intermediate	4 weeks AV + 1 cycle carbo/etoposide	Stromal type
85	F	2013	WT	primary	intermediate	4 weeks AV	Mixed type (blastema/epithelium)
86	M	2016	WT	primary	n/a[#]	untreated	Favorable histology WT, epithelial predominant
88	M	2015	WT	primary	intermediate	4 weeks AV	Stromal type
94	F	2016	WT	primary	intermediate	4 weeks AV	Mixed type (65% blastema)
95	M	2014	WT (Beckwith Wiedemann)	primary	unknown	4 weeks AV	Blastemal predominant
96	M	2014	WT	primary	unknown	4 weeks AV	Blastemal predominant
97	F	2013	WT	primary	intermediate	4 weeks AV	Regressive
98	F	2012	WT (Beckwith Wiedemann)	primary	high	8 weeks AV	Bilateral, material from 1 kidney. Diffuse anaplasia
101	F	2014	WT	primary	intermediate	6 weeks VAD	Stromal/epithelium
102	F	2016	Nephrogenic rest (Beckwith Wiedemann)	primary	n/a	4 weeks AV	n/a
103	F	2017	MRTK	primary	n/a	untreated	n/a
107	F	2002	RCC	primary	n/a	untreated	pX11.2 translocation
108	M	2000	RCC	primary	n/a	1 week sunitinib	n/a
109	F	2016	WT	primary	n/a[#]	untreated	Favorable histology WT, all three components present
119	M	2005	Relapsed Wilms[*]	Met (liver)	high	untreated	Metastasis with predominantly blastema
PMC-MRTK1	F	2015	MRTK	Met (lymph node)	n/a	6 weeks VAD + 1 additional VCR	n/a
CMN010T	M	2018	CMN	primary	n/a	untreated	Classic type
CMN012T	F	2018	CMN	primary	n/a	untreated	Mixed type
WT003T	M	2016	WT (cystic)	primary	n/a	untreated	Favorable histology WT, all three components present

* Tumor was a second relapse of a WT. Complete remission was reached after chemotherapy treatment of the primary tumor (ACT-D, VCR, DOX, radiotherapy) and of the first relapse (ifosfamide, cyclofosfamide, carboplatin, ETO). Complete remission was again reached by treating the second relapse with VCR and irinotecan and subsequently with ifosfamide, cyclofosfamide, carboplatin, ETO, high dose melphalan, radiotherapy. # According to the SIOP classification, untreated Wilms tumors are classified intermediate risk or higher (unless it is a CMN or CPDN).

Supplementary Table 1. Characteristics of the study participants.

Drugs	Target	Company	Product number
(+)-JQ-1	BRD4	Bioconnect/MedChem Express	HY-13030
Abemaciclib mesylate	CDK4; CDK6	Bioconnect/Selleck Chemicals	S7158
Afatinib	EGFR; HER2	Bioconnect/Selleck Chemicals	S1011
Alectinib	ALK	MTA-2015-0503/Roche/Chugai	N/A
Alisertib	Aurora A kinase	Bioconnect/MedChem Express	HY-10971
AMG 337	c-Met	Bioconnect/MedChem Express	HY-18696
Apatinib mesylate	VEGFR2	Bioconnect/Selleck Chemicals	S2221
AT-406	cIAP1-BIR3; cIAP2-BIR3; XIAP-BIR3	Bioconnect/Selleck Chemicals	S2754
AT7519	pan-CDK	Astex	N/A
Axitinib	VEGFR1; VEGFR2; VEGFR3; PDGFR-β; c-Kit	Bioconnect/Selleck Chemicals	S1005
AZD1775	WEE1	Bioconnect/MedChem Express	HY-10993
AZD4547	FGFR1; FGFR2; FGFR3; FGFR4; KDR	Bioconnect/MedChem Express	HY-13330
AZD5582	cIAP1; cIAP2; XIAP	Bioconnect/MedChem Express	HY-12600
AZD6738	ATR	Bioconnect/Selleck Chemicals	S7693
AZD8055	mTORC1; mTORC2	Bioconnect/Selleck Chemicals	S1555
BCT-100	Arginase	MTA-01211/Bio-Cancer Treatment Int	N/A
BIBR 1532	TERT	Bioconnect/Selleck Chemicals	S1186
Binimetinib	MEK1; MEK2	Bioconnect/Selleck Chemicals	S7007
Birinapant	cIAP1; XIAP	Bioconnect/Selleck Chemicals	S7015
Bortezomib	Proteasome	Bioconnect/MedChem Express	HY-10227
Buparlisib	PI3Kα; PI3Kβ; PI3Kδ; PI3Kγ	Bioconnect/Selleck Chemicals	S2247
Cabozantinib S-malate	MET; VEGFR; AXL; RET; KIT	Bioconnect/Selleck Chemicals	S4001
Capmatinib	c-Met	Bioconnect/MedChem Express	HY-13404
CC122	DNA-PK	Bioconnect/MedChem Express	HY-100507
Cediranib	VEGFR(KDR); Flt1; Flt4; c-Kit; PDGFRβ	Bioconnect/Selleck Chemicals	S1017
Ceritinib	ALK	Bioconnect/Selleck Chemicals	S7083
Cobimetinib	MEK1	Bioconnect/MedChem Express	HY-13064
CPI-455	KDM5A; KDM5B; KDM5C	Bioconnect/MedChem Express	HY-100421
Crenolanib	PDGFRα; PDGFRβ; FLT3	Bioconnect/MedChem Express	HY-13223
Crizotinib	c-Met; ALK	Bioconnect/Selleck Chemicals	S1068
CUDC-907	PI3K; HDAC	Bioconnect/MedChem Express	HY-13522
Dabrafenib	BRAFV600E	Bioconnect/MedChem Express	HY-14660
Dasatinib	Abl; Src; c-Kit	Bioconnect/MedChem Express	HY-10181
Decitabine	DNA methyltransferase	Bioconnect/Selleck Chemicals	S1200
Defactinib	FAK	Bioconnect/MedChem Express	HY-12289
Dovitinib	Flt3; c-Kit; FGFR1; FGFR3; VEGFR1; VEGFR2; VEGFR3; VEGFR4	Bioconnect/MedChem Express	HY-50905
EHT 1864 (2HCL)	Rac1; Rac1b; Rac2; Rac3	Bioconnect/MedChem Express	HY-16659
Ensartinib	ALK	Bioconnect/MedChem Express	HY-16590
Entinostat	HDAC1; HDAC3	Bioconnect/Selleck Chemicals	S1053
Entospletinib	SYK	Bioconnect/MedChem Express	HY-15968
Entrectinib	TrkA; TrkB; TrkC; ROS1; ALK	Bioconnect/MedChem Express	HY-12678
Epidaza	HDAC	Bioconnect/MedChem Express	HY-13592
Erlotinib hydrochloride	EGFR	Bioconnect/MedChem Express	HY-12008
Everolimus	mTORC1	Bioconnect/Selleck Chemicals	S1120
EW-7197	ALK4; ALK5	Bioconnect/MedChem Express	HY-19928
FX1	BCL6	Bioconnect/MedChem Express	HY-102027/CS-7688
Galunisertib	ALK5	Bioconnect/Selleck Chemicals	S2230
Ganetespib	HSP90	Bioconnect/Selleck Chemicals	S1159
Glasdegib	SMO	Bioconnect/MedChem Express	HY-16391
GSK J4 hydrochloride	JMJD3; UTX	Bioconnect/Selleck Chemicals	S7070
GSK1070916	Aurora B kinase; Aurora C kinase	Bioconnect/Selleck Chemicals	S2740
GSK2636771	PI3Kβ	Bioconnect/Selleck Chemicals	S8002
GSK269962A	ROCK1; ROCK2	Bioconnect/MedChem Express	HY-15556
GSK461364	PLK1	Bioconnect/Selleck Chemicals	S2193
I-BET-762	BRD2; BRD3; BRD4	Bioconnect/MedChem Express	HY-13032
I-BRD9	BRD9	Bioconnect/MedChem Express	HY-18975
Ibrutinib	BTK	Bioconnect/Selleck Chemicals	S2680
Icotinib	EGFR	Bioconnect/Selleck Chemicals	S2922
Idasanutlin	p53-MDM2	MTA/Roche	N/A
Imatinib mesylate	v-Abl; c-Kit; PDGFR	Bioconnect/Selleck Chemicals	S1026
Ipatasertib	AKT1; AKT2; AKT3	MTA-OR-214920/Genentech	Barcode: B000823882
IPI549	PI3Kγ	Bioconnect/MedChem Express	HY-100716
KU-55933	ATM	Bioconnect/Selleck Chemicals	S1092
KU-60019	ATM	Bioconnect/Selleck Chemicals	S1570
Lapatinib	EGFR; HER2	Bioconnect/MedChem Express	HY-50898
Larotrectinib sulfate	Trk receptor	Bioconnect/Selleck Chemicals	S7960
Lenvatinib	VEGFR2; VEGFR3; VEGFR1; FGFR1; PDGFRα; PDGFRβ	Bioconnect/MedChem Express	HY-10981
LGK974	PORCN	Bioconnect/MedChem Express	HY-17545
Linsitinib	IGF-1R	Bioconnect/Selleck Chemicals	S1091
LMK-235	HDAC4; HDAC5	Bioconnect/MedChem Express	HY-18998
Lorlatinib	ALK; ROS1	Bioconnect/Selleck Chemicals	S7536
LTURM34	DNA-PK	Bioconnect/MedChem Express	HY-101667
LY3023414	PI3Kα; PI3Kβ; PI3Kδ; PI3Kγ; mTORC1; mTORC2; DNA-PK	Bioconnect/MedChem Express	HY-12513

Drugs	Target	Company	Product number
LY3039478	NOTCH	Bioconnect/MedChem Express	HY-12449
Masitinib	c-Kit; PDGFRα; PDGFRβ	Bioconnect/MedChem Express	HY-10209
Merestinib	c-Met	Bioconnect/MedChem Express	HY-15514
Miransertib	AKT1; AKT2; AKT3	Bioconnect/MedChem Express	HY-19719
MK-2206 dihydrochloride	AKT1; AKT2; AKT3	Bioconnect/Selleck Chemicals	S1078
MLN 4924	Nedd8-activating enzyme	Bioconnect/Axon Medchem	2038
MM-102	MLL1	Bioconnect/Selleck Chemicals	S7265
Momelotinib	JAK1; JAK2; JAK3	Bioconnect/Selleck Chemicals	S2219
MSC2490484A	DNA-PK	Bioconnect/MedChem Express	HY-101570/CS-0021723
Mubritinib	HER2	Bioconnect/Selleck Chemicals	S2216
MX69	MDM2/XIAP	Bioconnect/MedChem Express	HY-100892
Navitoclax	BCL-2; BCL-XL; BCL-W	Bioconnect/Selleck Chemicals	S1001
Neratinib	HER2; EGFR; weakly KDR; weakly Src	Bioconnect/MedChem Express	HY-32721
Nilotinib	Bcr-Abl	Bioconnect/Selleck Chemicals	S1033
Niraparib	PARP1; PARP2	Bioconnect/MedChem Express	HY-10619
NVP-HDM201	MDM2	Bioconnect/MedChem Express	HY-18658/CS-7654
Olaparib	PARP1; PARP2	Bioconnect/Selleck Chemicals	S1060
OTX-015	BRD2; BRD3; BRD4	Bioconnect/MedChem Express	HY-15743
Palbociclib hydrochloride	CDK4; CDK6	Bioconnect/Selleck Chemicals	S1116
Panobinostat	pan-HDAC	Bioconnect/Selleck Chemicals	S1030
Pazopanib	VEGFR1; VEGFR2; VEGFR3; PDGFRα; PDGFRβ; FGFR1; c-Kit; c-FMS	Bioconnect/MedChem Express	HY-10208
PCI-34051	HDAC8	Bioconnect/Selleck Chemicals	S2012
PD-0325901	MEK1; MEK2	Bioconnect/Selleck Chemicals	S1036
PD-1/PD-L1 inhibitor 1	PD-1; PD-L1	Bioconnect/MedChem Express	HY-19991
Perifosine	AKT1; AKT2; AKT3	Bioconnect/MedChem Express	HY-50909
Pexidartinib	CSF-1R; Kit; Flt3	Bioconnect/MedChem Express	HY-16749
PF-06651600	JAK3	Sigma-Aldrich	PZ0316
Pictilisib	PI3Kα; PI3Kδ	MTA-OR-214920/Genentech	Barcode: B000824090
Pinometostat	DOT1L	Bioconnect/MedChem Express	HY-15593
Plerixafor	Chemokine receptor antagonist for CXCR4 and CXCL12-mediated chemotaxis	Bioconnect/MedChem Express	HY-10046
Ponatinib	VEGFR2; Bcr-Abl; PDGFRα; FGFR1	Bioconnect/Selleck Chemicals	S1490
Prexasertib	CHK1	Bioconnect/MedChem Express	HY-18174
Quizartinib	Flt3	Bioconnect/Selleck Chemicals	S1526
Ravoxertinib	ERK	MTA-OR-214920/Genentech	Barcode: B000847422
Regorafenib	VEGFR1; VEGFR2; VEGFR3; PDGFRβ; Kit; RET; Raf-1	Bioconnect/MedChem Express	HY-10331
RG6146	BRD2; BRD3; BRD4; BRDT	Bioconnect/MedChem Express	HY-15846
Ribociclib	CDK4; CDK6	Bioconnect/MedChem Express	HY-15777
Romidepsin	HDAC1; HDAC2	Bioconnect/MedChem Express	HY-15149
Rucaparib phosphate	PARP1; PARP2	Bioconnect/Selleck Chemicals	S1098
Ruxolitinib	JAK1; JAK2	Bioconnect/MedChem Express	HY-50856
S63845	MCL-1	Active Biochem	A-6044
Sapitinib	EGFR; HER2; HER3	Bioconnect/Selleck Chemicals	S2192
SAR405	VPS34	Bioconnect/MedChem Express	HY-12481
Saracatinib	Src; c-YES; FYN; LYN; BLK; FGR; LCK; Abl; EGFR	Bioconnect/Selleck Chemicals	S1006
Savolitinib	c-Met	Bioconnect/MedChem Express	HY-15959
Selinexor	CRM1	Bioconnect/Selleck Chemicals	S7252
Selumetinib	MEK1	Bioconnect/Selleck Chemicals	S1008
SHP099 hydrochloride	SHP2	Bioconnect/MedChem Express	HY-100388A
Sirolimus	mTORC1	Bioconnect/MedChem Express	HY-10219
Sonidegib	SMO	Bioconnect/Selleck Chemicals	S2151
Sorafenib	Raf-1; B-Raf; VEGFR-2	Bioconnect/MedChem Express	HY-10201
Sunitinib	VEGFR2; PDGFRβ; c-Kit	Bioconnect/MedChem Express	HY-10255A
Talazoparib	PARP1; PARP2	Bioconnect/Selleck Chemicals	S7048
Tanespimycin	HSP90	Bioconnect/Selleck Chemicals	S1141
Taselisib	PI3Kα mutant	MTA-OR-214920/Genentech	Barcode: B000824007
Tazemetostat	EZH2	Bioconnect/MedChem Express	HY-13803
Temsirolimus	mTORC1	Bioconnect/MedChem Express	HY-50910
TH1579	MTH1	MTA-01180/Karolinska Institute	N/A
Tivantinib	c-Met	Bioconnect/MedChem Express	HY-50686
Tofacitinib citrate	JAK1; JAK2; JAK3	Bioconnect/MedChem Express	HY-40354A
Trametinib	MEK1; MEK2	Bioconnect/Selleck Chemicals	S2673
Vandetanib	VEGFR2; VEGFR3; EGFR	Bioconnect/MedChem Express	HY-10260
Varlitinib	EGFR; HER2	Bioconnect/MedChem Express	HY-10530
VE-822	ATR	Bioconnect/MedChem Express	HY-13902
Vemurafenib	BRAFV600E	MTA-OR-214920/Genentech	Barcode: B000847423
Venetoclax	BCL-2	Bioconnect/Selleck Chemicals	S8048
Vismodegib	SMO	Bioconnect/Selleck Chemicals	S1082
Vistusertib	mTORC1; mTORC2	Bioconnect/MedChem Express	HY-15247
Volasertib	PLK1	Bioconnect/Selleck Chemicals	S2235
Vorinostat	pan-HDAC	Bioconnect/MedChem Express	HY-10221
XAV-939	TNKS1; TNKS2	Bioconnect/Selleck Chemicals	S1180
YM155	Survivin	Bioconnect/Selleck Chemicals	S1130
YO-01027	γ-secretase	Bioconnect/MedChem Express	HY-13526

Supplementary Table 2. List targeted compounds tested in high throughput drug screenings of Wilms tumour organoid cultures.
