# Supplementary material for: An organoid biobank for childhood kidney cancers that captures disease and tissue heterogeneity
Source: Nat Commun. 2020 Mar 11;11:1310. doi: 10.1038/s41467-020-15155-6 (PMC7066173; doi:10.1038/s41467-020-15155-6)
Supplement: Supplementary file 2 — Description of Additional Supplementary Files [file 41467_2020_15155_MOESM2_ESM.pdf]

Description of Additional Supplementary Files

File Name: Supplementary Data 1
Description: List of differentially expressed genes for all organoids and clusters.
Differential expression data obtained for all clusters as well as all individual organoids. Positive markers for each cell population are shown in separate sheets labelled accordingly. Indicated in each table are the gene symbol, pvalue, average log fold change difference between the two compared groups, percentage of cells expressing the gene in group 1, percentage of cells expressing the gene in group 2, Bonferroni corrected p-value. Statistical analysis have been performed using the Wilcoxon test two-sided with 1.8-fold expression cut-off and 5% Bonferroni multiple testing corrected statistical significance cut-off.

File Name: Supplementary Data 2
Description: Variant Allele Frequency (VAF).
Indicated are mutation type, CNA, and respective VAF.

File Name: Supplementary Movie 1
Description: High resolution 3D imaging of 51T Wilms tumour organoids. E-cadherin (red), SIX2 (green) and CD90 (white).

File Name: Supplementary Movie 2
Description: High resolution 3D imaging of 88T Wilms tumour organoids. E-cadherin (red), CD90 (white), DAPI (blue)
