# Supplementary material for: An organoid biobank for childhood kidney cancers that captures disease and tissue heterogeneity
Source: Nat Commun. 2020 Mar 11;11:1310. doi: 10.1038/s41467-020-15155-6 (PMC7066173; doi:10.1038/s41467-020-15155-6)
Supplement: Supplementary file 7 — Reporting Summary [file 41467_2020_15155_MOESM7_ESM.pdf]

nature research

Corresponding author(s): Jarno Drost

Last updated by author(s): 24-01-2020

Reporting Summary

Nature Research wishes to improve the reproducibility of the work that we publish. This form provides structure for consistency and transparency in reporting. For further information on Nature Research policies, see Authors & Referees and the Editorial Policy Checklist.

Statistics

For all statistical analyses, confirm that the following items are present in the figure legend, table legend, main text, or Methods section.

n/a	Confirmed	
☐	☒	The exact sample size (n) for each experimental group/condition, given as a discrete number and unit of measurement
☐	☒	A statement on whether measurements were taken from distinct samples or whether the same sample was measured repeatedly
☐	☒	The statistical test(s) used AND whether they are one- or two-sided<br>*Only common tests should be described solely by name; describe more complex techniques in the Methods section.*
☒	☐	A description of all covariates tested
☐	☒	A description of any assumptions or corrections, such as tests of normality and adjustment for multiple comparisons
☐	☒	A full description of the statistical parameters including central tendency (e.g. means) or other basic estimates (e.g. regression coefficient) AND variation (e.g. standard deviation) or associated estimates of uncertainty (e.g. confidence intervals)
☐	☒	For null hypothesis testing, the test statistic (e.g. F, t, r) with confidence intervals, effect sizes, degrees of freedom and P value noted<br>*Give P values as exact values whenever suitable.*
☒	☐	For Bayesian analysis, information on the choice of priors and Markov chain Monte Carlo settings
☐	☒	For hierarchical and complex designs, identification of the appropriate level for tests and full reporting of outcomes
☐	☒	Estimates of effect sizes (e.g. Cohen's d, Pearson's r), indicating how they were calculated

Our web collection on statistics for biologists contains articles on many of the points above.

Software and code

Policy information about availability of computer code

Data collection	See below for software used.
Data analysis	GraphPad Prism v7.04<br>Imaris software v.9.2.1<br>ImageJ v.1.50e<br>Kaluza Analysis 2.1<br>Burrows-Wheeler Aligner v0.7.5a<br>Sambamba v0.6.8<br>Analysis Toolkit (GATK) IndelRealigner v3.4-46<br>GATK HaplotypeCaller v3.4-46<br>Single Nucleotide Polymorphism Database v137.b37<br>STAR version 2.6.1<br>featureCounts version 1.5.2<br>R package Seurat (version 3.0.2)<br>R package clusterProfiler version 3.12<br>SingleR version 1.0.1<br>STAR-fusion (version 1.4.0)<br>Chimeraviz R package (version 3.8)

For manuscripts utilizing custom algorithms or software that are central to the research but not yet described in published literature, software must be made available to editors/reviewers. We strongly encourage code deposition in a community repository (e.g. GitHub). See the Nature Research guidelines for submitting code & software for further information.

Data

Policy information about availability of data

All manuscripts must include a data availability statement. This statement should provide the following information, where applicable:

- Accession codes, unique identifiers, or web links for publicly available datasets
- A list of figures that have associated raw data
- A description of any restrictions on data availability

The sequencing data have been deposited to the European Genome-Phenome Archive (www.ebi.ac.uk/ega/) under accession numbers EGAD00001005319 [https://www.ebi.ac.uk/ega/search/site/EGAD00001005319] and EGAD00001005318 [https://www.ebi.ac.uk/ega/search/site/EGAD00001005318]. DNA methylation data have been deposited to GEO (www.ncbi.nlm.nih.gov/geo/) under accession number GSE137544 [https://www.ncbi.nlm.nih.gov/geo/query/acc.cgi?acc=GSE137544]. COSMIC SigProfiler database [https://www.synapse.org/#!Synapse:syn11967914] has been used for mutational signatures analysis. Filtering scripts used mutational signatures analysis are available at https://github.com/UMCUGenetics/SNVFI and https://github.com/ToolsVanBox/INDELFI.

Field-specific reporting

Please select the one below that is the best fit for your research. If you are not sure, read the appropriate sections before making your selection.

☒ Life sciences ☐ Behavioural & social sciences ☐ Ecological, evolutionary & environmental sciences

For a reference copy of the document with all sections, see nature.com/documents/nr-reporting-summary-flat.pdf

Life sciences study design

All studies must disclose on these points even when the disclosure is negative.

Sample size	All patients with pediatric kidney tumors that have been treated at the Princess Maxima Center and have given informed consent have been included in the study (54)
Data exclusions	No data was excluded
Replication	For each of the experiments, we specify in the legends the number of replicates conducted.
Randomization	Not relevant as no comparisons were made between different experimental groups.
Blinding	All analyses were performed blinded. For instance, H&E stainings were examined by the pathologist without providing any patient information (e.g. tumor type, stage, etc.).

Reporting for specific materials, systems and methods

We require information from authors about some types of materials, experimental systems and methods used in many studies. Here, indicate whether each material, system or method listed is relevant to your study. If you are not sure if a list item applies to your research, read the appropriate section before selecting a response.

Materials & experimental systems

n/a	Involved in the study	
☐	☒	Antibodies
☒	☐	Eukaryotic cell lines
☒	☐	Palaeontology
☒	☐	Animals and other organisms
☐	☒	Human research participants
☒	☐	Clinical data

Methods

n/a	Involved in the study	
☒	☐	ChIP-seq
☐	☒	Flow cytometry
☒	☐	MRI-based neuroimaging

Antibodies

Antibodies used	The following primary antibodies were used for immunohistochemical staining: desmin (Leica Novacastra, NCL-L-Des-Derll, 1:100), INI-1 (BD Transduction Laboratories, 612111, 1:400), P53 (Dako, M7001, 1:6000). The following antibodies were used for immunofluorescent stainings: SIX2 (Proteintech, 11562-1-AP, 1:200), E-cadherin clone ECCD-2 (Thermo Fisher, 13-1900, 1:200/1:500), CD90 clone EPR3133 (Abcam, 133350, 1:100), CD90-APC clone 5E10 (Biolegend, 328113, 1:200). The following antibodies were used for FACS: Alexa-fluor 488 anti-human CD326 EPCAM clone 9C4 (Bio Legend, 324210, 1:20), CD90-APC clone 5E10 (Biolegend, 328113, 1:50). The following antibodies were used for Western Blot: P53 clone DO-1 (sc-126, Santa Cruz Biotechnology, 1:1000), GAPDH (ab-9485, Abcam,1:1000).
Validation	Antibodies were validated for use in these systems by the manufacturers who have provided references on each of their

websites using the catalog numbers provided above.
Proteintech: https://www.ptglab.com/products/SIX2-Antibody-11562-1-AP.htm#validation
Thermo Fisher: https://www.thermofisher.com/antibody/product/E-cadherin-Antibody-clone-ECCD-2-Monoclonal/13-1900
Abcam: https://www.abcam.com/cd90--thy1-antibody-epr3133-ab133350-references.html#top-500
https://www.abcam.com/gapdh-antibody-loading-control-ab9485.html
Biolegend: https://www.biolegend.com/fr-fr/products/apc-anti-human-cd90-thy1-antibody-4116
https://www.biolegend.com/fr-fr/products/alexa-fluor-488-anti-human-cd326-epcam-antibody-3759
Santa Cruz Biotechnology: https://www.scbt.com/p/p53-antibody-do-1
Antibodies were validated by including positive and negative control tissues.

Human research participants

Policy information about studies involving human research participants

Population characteristics	We collected tumors (and matching healthy kidney) from all pediatric patients (age < 18) with kidney tumors entering the Princess Maxima Center. We included in this study male and female patients with different diagnosis, with primary and/or metastatic tumors, patient that underwent different therapy regimens. All details about patients included in the study are mentioned in Supplementary Table 1.
Recruitment	All children treated in our institute for a renal tumor and signed informed consent was obtained were included.
Ethics oversight	All experiments with human tissue were approved by the medical ethical committee of the Erasmus Medical Center (Rotterdam, the Netherlands).

Note that full information on the approval of the study protocol must also be provided in the manuscript.

Flow Cytometry

Plots

Confirm that:

☒ The axis labels state the marker and fluorochrome used (e.g. CD4-FITC).

☒ The axis scales are clearly visible. Include numbers along axes only for bottom left plot of group (a 'group' is an analysis of identical markers).

☒ All plots are contour plots with outliers or pseudocolor plots.

☒ A numerical value for number of cells or percentage (with statistics) is provided.

Methodology

Sample preparation	MRTK and Wilms tumour organoids were dissociated into single-cell suspensions using TrypLE Express (Thermo Fisher) supplemented with Rho-kinase inhibitor Y-27632 (10 µM, Abmole). Single-cell suspensions were stained using mouse Alexa-fluor 488 anti-human CD326 EPCAM clone 9C4(1:20, Biolegend) and CD90-APC clone 5E10 (Biolgend, 328113, 1:50) as described.
Instrument	EPCAM-positive and -negative populations were separated using BD FACSAria - Fusion sorter (BD Biosciences). EPCAM positive and CD90 positive populations were separated using MoFlow Astrios (Beckman Coulter).
Software	Kaluza Analysis 2.1
Cell population abundance	After sorting, both populations were plated in BME and cultured as described in Methods section.
Gating strategy	In contrast to normal kidney tissue, MRTKs do not show epithelial differentiation. Therefore, we separated MRTK cells from contaminating normal kidney cells based on expression of the epithelial marker EPCAM. As for CD90 and EPCAM gating in Wilms tumor organoids, we used unlabelled cells of interest, single-color controls (UltraComp beads ThermoFisher, or partially killed cells of interest) followed by automatic compensation matrix calculation s(fluorescence-minus-one (FMO) controls for each marker).

☒ Tick this box to confirm that a figure exemplifying the gating strategy is provided in the Supplementary Information.
